# BIN2 inhibition suppress ovarian cancer progression meanwhile protect ovarian function through downregulating HDAC1 and RPS6 phosphorylation respectively

**DOI:** 10.1002/ctm2.70051

**Published:** 2024-10-16

**Authors:** Cong‐Rong Li, Shi‐Ya Xie, Shu‐Ping Zhang, Yan‐Jie Yang, Li‐Li Yang, Ying Cao, Li‐Li Wang, Feng‐Yu Zhu, Ruo‐Lei Wang, Zhi‐Xia Yang, Chen‐Chen Cui, Yan‐Ru Li, Jia‐Ning Xu, Feng Yue, Pei‐Zhe Tian, Qian Wang, Hong‐Jie Yao, Yi‐Chun Guan, Shao‐Di Zhang, Xiao‐Yan Ying, Dong Zhang, Cui‐Lian Zhang

**Affiliations:** ^1^ Department of Gynaecology and Obstetrics the First Affiliated Hospital of Anhui Medical University Hefei Anhui China; ^2^ State Key Lab of Reproductive Medicine and Offspring Health Nanjing Medical University Nanjing Jiangsu China; ^3^ NHC Key Laboratory of Study on Abnormal Gametes and Reproductive Tract Anhui Medical University Hefei Anhui China; ^4^ Key Laboratory of Population Health Across Life Cycle (Anhui Medical University) Ministry of Education of the People’s Republic of China Hefei Anhui China; ^5^ Department of Gynaecology and Obstetrics the Second Affiliated Hospital of Nanjing Medical University Nanjing Jiangsu China; ^6^ Reproductive Medical Center Henan Provincial People’s Hospital & People’s Hospital of Zhengzhou University Zhengzhou Henan China; ^7^ Guangzhou National Laboratory University of Chinese Academy of Sciences Guangzhou Guangdong China; ^8^ Center for Reproductive Medicine the Third Affiliated Hospital of Zhengzhou University Zhengzhou Henan China

Dear Editor,

Ovarian cancer, OC, covers approximately 314 000 cases with 66% mortality rate and 49% 5‐year survival rate in 2020.^1^ Bridge integrator 2 (BIN2) is correlated with the genesis and progression of various types of tumour including OC.^2^, ^3^, ^4^ Our analysis of GEPIA data showed that Bin2 transcripts were upregulated in OC and positively correlated with survival (Figure 1A and B). We verified the upregulation of BIN2 and phosphorylated BIN2 (p‐BIN2‐T423 and S424) in 60% of the clinical OC samples we collected (Figure 1C and D) and human A2780 OC cells (Figure S1A–C) and OC tissue microarrays (Figure S1E and 1F). However, BIN2 has not been investigated in OC. Previously, we showed that downregulating p‐BIN2 via competitive peptide BPP improved primordial follicle (PMF) and oocyte quality by downregulating p‐RPS6 and upregulating NNT in healthy, chemotherapy‐treated, and aging mice.^5^ Therefore, could BIN2 inhibition exert a ‘dual function’ – suppressing tumour progression while protecting ovarian function during OC genesis or progression, thereby safely alleviating young OC females?

**FIGURE 1:**
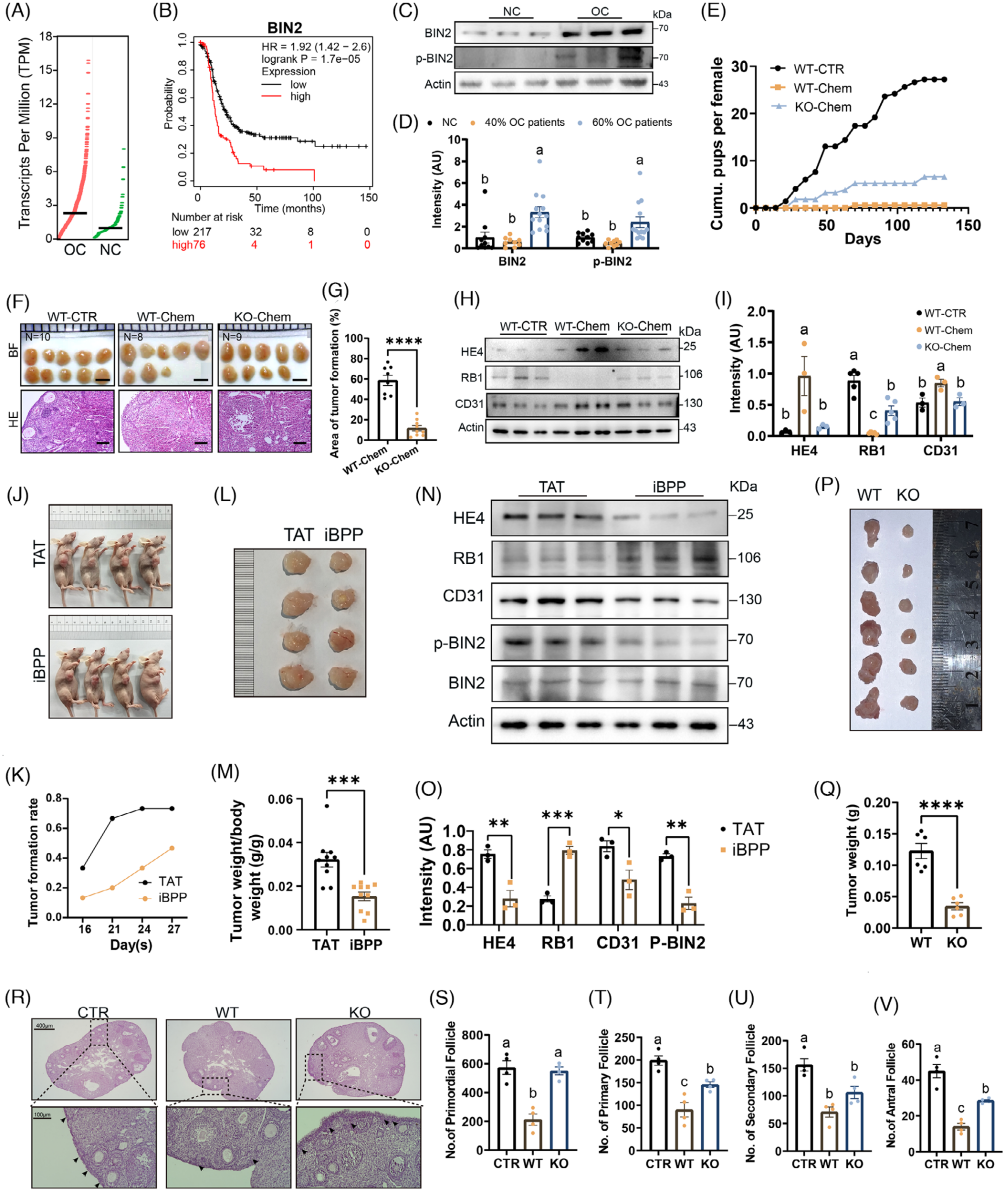
BIN2 inhibition or *Bin2* knockout suppresses ovarian cancer progression and improves female fertility. (A) Analysis of BIN2 expression data in GEPIA database showed that the BIN2 mRNA level was higher in OC (ovarian cancer) tissues than in normal control (NC) ovaries. *n* = 426 for OC group and *n* = 88 for NC group. (B) Correlation analysis with Kapian‐meier plotter showed that patients with high‐level BIN2 had lower progression‐free survival (PFS) rate in grade I and grade II ovarian cancer (GSE14764, *n* = 293). (C, D) Blot and quantification showed that BIN2 and p‐BIN2 was significantly upregulated in 60% of OC tissues collected from 22 OC patients. *N* = 10 for NC group. One‐way ANOVA. For BIN2, NC vs. 60% OC patients, **p = .0023; 40% OC patients vs. 60% OC patients, ***p = .0005. For p‐BIN2, NC vs. 60% OC patients, *p = .0225; 40% OC patients vs. 60% OC patients, **p = .0022. E‐I: WT‐CTR, WT B6 mice treated with PBS; WT‐chem, WT B6 mice treated with chemocarcinogen; KO‐chem, *Bin2*‐KO B6 mice treated with chemocarcinogen. (E) We used DMBA plus NMU to induce in situ tumourigenesis within ovaries. Assays of cumulative litter size (*n* = 5) showed that under in situ chemicarcinogen treatment, WT mouse almost completely lost fertility, whereas *Bin2‐*KO ovaries kept partially fertile. (F, G) Bright‐field image and quantification showed that the area coverage rate of transformed white ovarian tissue (transformed ovarian area / total ovarian area) was significantly larger in WT‐Chem group but much smaller in KO‐Chem group; HE staining of Paraffin section showed the tumour‐like white tissue lost features of ovarian structure. Scale bars in F, 2 mm for the upper line, 100 µm for the lower line. *N* = 10, 8, 9 for WT‐CTR, WT‐Chem, and KO‐Chem groups respectively. Unpaired two‐tailed *t*‐test; ****p < .0001. (H, I) Western blot and quantification showed that the level of common tumour marker including CD31, RB1 and HE4 significantly increased or decreased in WT‐Chem group but had much less change in KO‐Chem group. *N* = 3 for all groups; one‐way ANOVA. For HE4, WT‐CTR vs. WT‐Chem, *p = .0319; WT‐Chem vs. KO‐Chem, *p = .0459. For RB1, WT‐CTR vs. WT‐Chem, ****p < .0001; WT‐CTR vs. KO‐Chem, ***p = .0007; WT‐Chem vs. KO‐Chem, **p = .0062. For CD31, WT‐CTR vs. WT‐Chem, *p = .0345; WT‐Chem vs. KO‐Chem, *p = .0427. (J–M) In A2780‐injected immunodeficient mice, iBPP treatment significantly decreased percentage of tumour formation (J and K) and tumour volume (L and M). *N* = 10 for both groups; unpaired two‐tailed *t*‐test; ***p = .0004. (N, O) Blot and quantification showed that the level of CD31, RB1, HE4 and p‐BIN2 significantly increased or decreased in tumours of iBPP group. *N* = 3 for both groups; unpaired two‐tailed *t*‐test. For HE4, **p = .0085. For RB1, ***p = .0006. For CD31, *p = .0385. For p‐BIN2, **p = .0019. (P, Q) ID8 OC cells were transplanted into sub‐axillary region of WT and *Bin2*‐KO B6 mice to induce ectopic OC formation, the tumour sizes and weights of *Bin2*‐KO mice were smaller than those of WT mice. *N* = 6 for both groups; Unpaired two‐tailed *t*‐test; ***p = .0003. (R–V) HE staining of ovary section and follicle counting (*n* = 4) showed that the number of primordial follicles and growing follicles at each stage were significantly higher in *Bin2*‐KO group than in WT group under ectopic OC invasion. The black arrows indicate primordial follicles. Scale bars, 400 µm for the upper line of R and 100 µm for the lower line of R. *N* = 4 for all groups. One‐way ANOVA. CTR, WT B6 mice treated with PBS; WT, WT B6 mice allografted with mouse ID8 OC cells; KO, *Bin2*‐KO B6 mice allografted with ID8 OC cells. For primordial follicle, CTR vs. WT, ***p = .0003; WT vs. KO, ***p = .0004. For primary follicle, CTR vs. WT, ***p = .0002; CTR vs. KO, *p = .0239; WT vs. KO, *p = .0193. For secondary follicle, CTR vs. WT, ***p = .0007; CTR vs. KO, *p = .021. For antral follicle, CTR vs. WT, ****p < .0001; CTR vs. KO, **p = .0025; WT vs. KO, **p = .0057. Different letters above columns in the statistical graphs indicate significant difference.

To address the upper assumption, we employed three OC models. Firstly, we used two chemocarcinogens, dimethylbenz[a]anthracene (DMBA) and methyl‐N‐nitrosourea (MNU), to induce in situ tumourigenesis within mouse ovaries. We found that BIN2 knockout partially recovered chemocarcinogen‐induced fertility loss (Figure 1E), meanwhile reduced ovarian transformed areas (Figure 1F and G), and recovered CD31 (blood vessel marker), HE4 (EOC marker), p‐RB1 (tumour suppressor), and Ki67 (proliferation marker) levels (Figures 1H and I and S2A–D). Secondly, we xenografted A2780 OC cells into immunodeficient mice. Inhibition of BIN2 phoshorylation by iBPP (improved BPP, Figure S3A), which was shown to effectively impede multiple indexes in human A2780 (Figure S3B–O) or H08910 (Figure S4A–I) cells but not normal ovarian epithelial cells, Moody (Figure S5A–I), significantly slowed OC progression in immunodeficient mice (Figure 1J–M). Increased RB1 and decreased CD31& HE4 supported these findings (Figure 1N and O). However, the first two models are unsuitable for ovarian function analyses as both chemocarcinogen‐treated and immunodeficient mice have low fertility. Therefore, thirdly, we used ID8 mouse OC‐cell allografts in normal B6 mice to analyse both OC progression and ovarian performance. BIN2 knockout (Figure 1P and Q) or inhibition (Figure S6A–E) significantly decreased OC progression or lung‐ward metastasis while restoring the numbers of primordial and developing follicles in mice (Figure 1R–V).

The expression and regulation patterns of many genes and proteins in OC vary from those in normal tissue. Therefore, the next urgent question is whether p‐RPS6 remains the key target of p‐BIN2 in OC. Surprisingly, we found no significantly positive correlation between p‐RPS6 and p‐BIN2 in clinical OC tissues (Figure 2A). Therefore, we guessed that BIN2 has other, more important targets in OC. We compared the interacting proteins of BIN2‐WT, inactive (S423A and T424A), and constitutively active (S423D and T424D) mutants through IP‐LCMS and found that only the active mutant drew HDAC1 (Figure 2B, red dot‐line rectangle). We verified the interaction between BIN2‐DD and HDAC1 via co‐IP (Figure 2C). Next, we found that the ratio of p‐HDAC1 to p‐RPS6 in OC was five‐fold higher than in healthy ovaries (Figure 2D and E), indicating that HDAC1 might dominate over RPS6 in binding BIN2 in OC tissues. HDAC1 is a deacetylase involved in tumourigenesis.^6^, ^7^ Furthermore, BIN2 inhibition significantly decreased p‐HDAC1‐S421 but not p‐HDAC2‐S424, suggesting BIN2's preference for HDAC1 (Figure 2F–I). Next, we showed that Sf9‐purified BIN2‐WT but not BIN2‐AA could dose‐dependently phosphorylate HDAC1‐S421 (Figure 2J–M). In addition, the purified BIN2‐DUF domain containing T423 and S424 but not BIN2‐BAR domain showed strong activity (Figure 2N–Q). Finally, immunohistochemistry on a human OC tissue chip showed a strong positive correlation between p‐BIN2 and p‐HDAC1 in identical OC tissues (Figure 2R and S).

**FIGURE 2:**
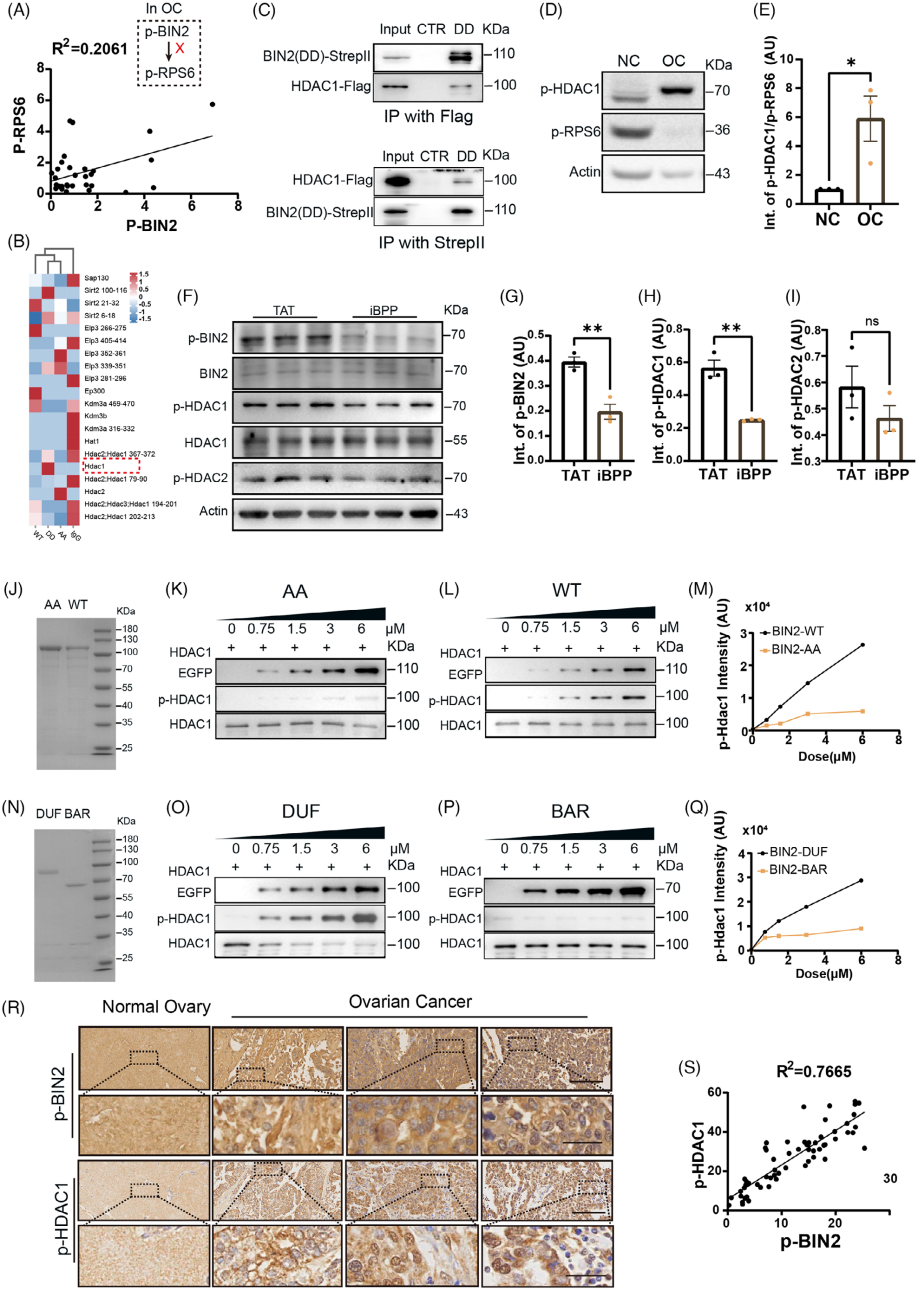
BIN2 directly binds and phosphorylates HDAC1. (A) Blot and quantification showed that there is no clear correlation between p‐BIN2 and p‐RPS6 in clinical OC samples (*n* = 31). (B) Immunoprecipitation with strep II antibody in BIN2‐WT, inactive (S423A & T424A) or constitutively active (S423D & T424D) mutant‐transfected A2780 cells and LC‐MS showed that only active mutant baited HDAC1(red dot‐line rectangle); however, none of BIN2 construct bait RPS6. (C) Bin2‐DD‐strep II and HDAC1‐flag constructs (in pcDNA3.1+) were co‐transfected into A2780 cells, co‐IP with strep II (lower) and flag (upper) showed that the active form (DD) of BIN2 interacts with HDAC1. (D, E) Blot and quantification showed that p‐HDAC1 level in OC is much higher than in ovaries, while p‐RPS6 level in tumour is much lower than in ovaries, as a result, the ratio of p‐HDAC1: p‐RPS6 in OC is over 6‐fold higher than that in ovaries. *N* = 3 for both groups; Unpaired two‐tailed *t*‐test; *p = .0352. (F–I) Blot and quantification showed that BIN2 inhibition by iBPP in A2780 cells significantly decreased p‐BIN2 (F and G) and p‐HDAC1 (S421) (F and H), but did not affect the level of p‐HDAC2 (F and I). *N* = 3 for both groups; unpaired two‐tailed *t*‐test. For p‐BIN2, **p = .0053. For p‐HDAC1, **p = .0029. (J) SDS‐PAGE and comassie staining of BIN2‐WT and ‐AA mutants (inactive form, S423A & T424A) purified from Sf9 cells showed the purity of these two proteins. (K–M) In vitro phosphorylation assays with proteins from J showed that BIN2‐WT increased p‐HDAC1 in a dose‐dependent manner, while BIN2‐AA mutant had much less activity on HDAC1 phosphorylation. (N) SDS‐PAGE and commasie staining of BIN2‐DUF and BIN2‐BAR purified from Sf9 cells showed the purity of these two BIN2 domains. (O–Q) In vitro phosphorylation assays with proteins from N showed that BIN2‐DUF increased p‐HDAC1 in a dose‐dependent manner, while BIN2‐BAR had much less activity on HDAC1 phosphorylation. (R, S) Immunohistochemistry and quantification of p‐BIN2 or p‐HDAC1 on the same region of the same tissue (but different Z sections) of a human ovary tissue chip showed that both p‐BIN2 and p‐HDAC1 are higher in OC tissues than in normal ovaries, and p‐BIN2 level is positively correlated with p‐HDAC1 level (*n* = 61). Scale bars for R, 100 µm. Scale bars in zoom for R, 25 µm.

The next key question was how p‐BIN2 promotes OC progression through p‐HDAC1. HDAC1 is reportedly the primary deacetylase for H3K27ac, and increased H3K27ac fostered the advancement of various types of tumour by promoting the transcription of TSGs (tumour suppressor genes).^8^, ^9^ We found that in either Bin2‐KO chemicarcinogen‐treated ovaries (Figure 3A–C) or iBPP‐treated OC cells (Figure 3D–F), H3K27ac, but not H3K27me3, was significantly upregulated. In addition, iBPP treatment did not affect H3K9ac but downregulated H3K14ac (Figure S7A and B). Further, chip‐seq showed that BIN2 inhibition significantly increased the binding of H3K27ac, but not H3K9ac or H3K14ac, with peaks 5 kb from the transcription start site (TSS) of multiple TSGs, such as Mbd4, Topors, and Sh2b3 (Figure 3G–J). The KEGG enrichment of H3K27ac binding regions was distinct from those of H3K9ac or H3K14ac and more tumour‐related (red dot‐line rectangle, Figure S7C–E). Accordingly, BIN2 inhibition significantly upregulated the expression of the upper TSGs (Figure 3K–M). Finally, HDAC1 overexpression could revert the iBPP‐induced p‐HDAC1 decrease and H3K27ac increase (Figure 3N–Q) and upregulation of the upper TSGs (Figure 3R–U). In addition, iBPP treatment didn't alter the transcription of multiple known cancer‐related genes including Brca1/2, Tp53, Pik3ca, and Kras (Figure S7F and G). These findings further support the p‐BIN2→p‐HDAC1→H3K27ac→TSGs axis.

**FIGURE 3:**
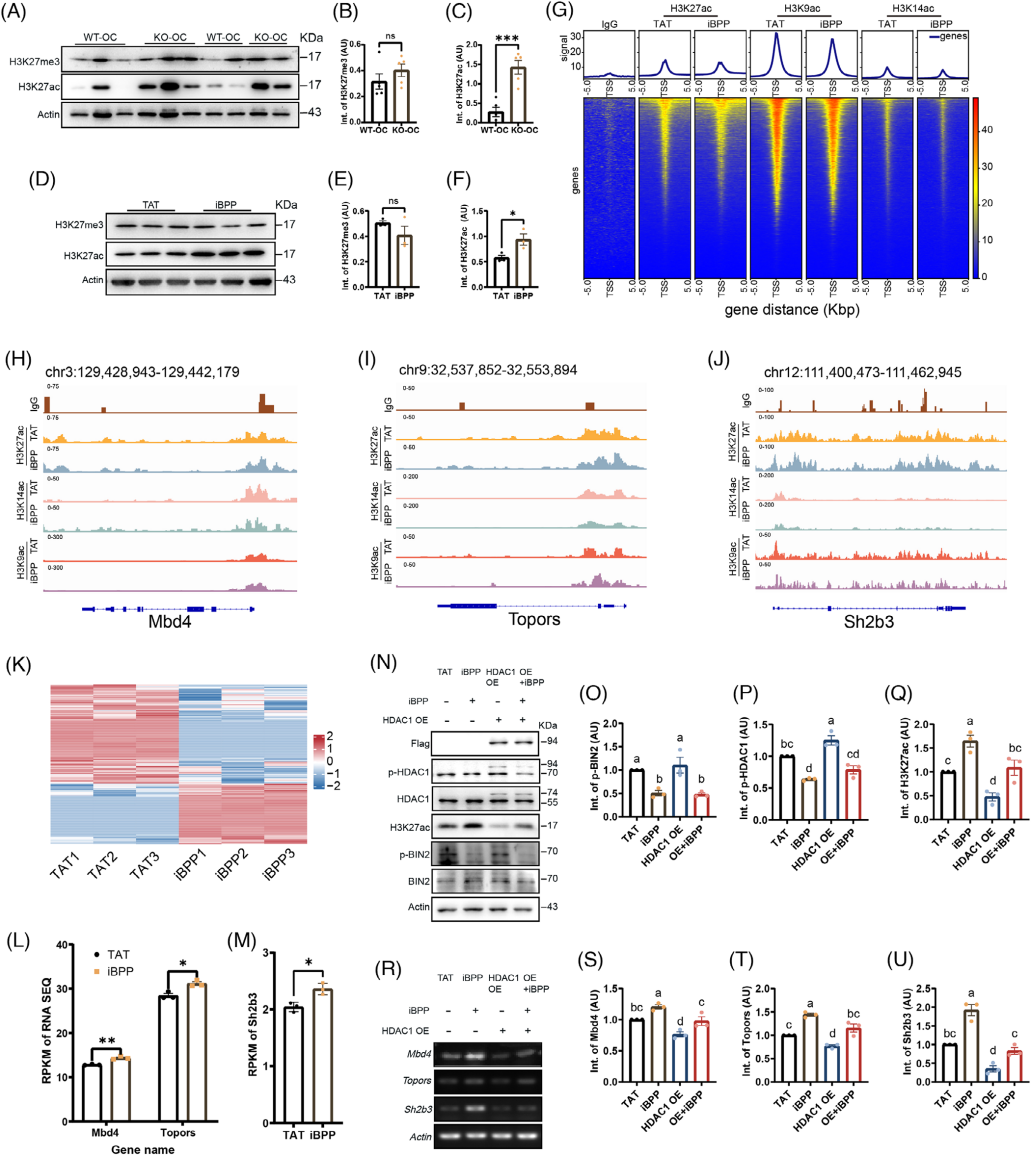
BIN2 inhibition upregulates TSGs transcription through p‐HDAC1→H3K27ac. (A–C) Blot and quantification showed that *Bin2* knockout significantly upregulate H3K27ac but did not affect H3K27me3 in chemicarcinogen‐treated ovaries. *N* = 5 for both groups; unpaired two‐tailed *t*‐test. For H3K27ac, ***p = .0007. (D–F) Blot and quantification showed that BIN2 inhibition by iBPP significantly upregulate H3K27ac but did not affect H3K27me3 in A2780 OC cells. *N* = 3 for both groups; unpaired two‐tailed *t*‐test. For H3K27ac, *p = .0418. (G) All‐gene intensity map of Cut & tag‐seq showed that BIN2 inhibition significantly increased H3K27ac (but not H3K9ac and H3K14ac) binding to the regions 5Kb from TSS (transcription start site) of multiple tumour suppressor genes (TSGs). (H–J) Intensity maps of H3K27ac, H3K14ac and H3K9ac binding to three representative TSGs, Mbd4, Topors, and Sh2b3. K. RNA sequencing heat map showed that at the threshold of |log2(BPP/CTR)| ≥ 1.2, there were 578 DEGs (differentially expressed genes) between CTR and iBPP‐treated A2780 OC cells. (L, M) Analysis of the representative RPKM values from RNA sequencing (K) showed that iBPP treatment significantly upregulated the expression levels of the upper (H–J) three TSGs. *N* = 3 for both groups; unpaired two‐tailed *t*‐test. For *Mbd4*, **p = .0019. For *Topors*, *p = .0113. For *Sh2b3*, *p = .014. N–Q. Blot and quantification showed that HDAC1 overexpression in A2780 cell could rescue iBPP‐induced p‐HDAC1 decrease and H3K27ac increase. TAT (TAT‐only peptide) acted as control. *N* = 3 for all groups. One‐way ANOVA. For p‐BIN2, TAT vs. iBPP, *p = .0196; TAT vs. OE+iBPP, *p = .0148; iBPP vs. HDAC1 OE, **p = .0068; HDAC1 OE vs. OE+iBPP, **p = .0052. For p‐HDAC1, TAT vs. iBPP, **p = .0035; TAT vs. HDAC1 OE, *p = .0277; iBPP vs. HDAC1 OE, ****p < .0001; HDAC1 OE vs. OE+iBPP, ***p = .0007. For H3K27ac, TAT vs. iBPP, *p = .0137; TAT vs. HDAC1 OE, *p = .039; iBPP vs. HDAC1 OE, ***p = .0003; iBPP vs. OE+iBPP, *p = .0293; HDAC1 OE vs. OE+iBPP, *p = .018. R‐U. Reverse Transcription PCR and quantification showed that HDAC1 overexpression in A2780 cell could rescue iBPP‐induced transcription upregulation of the upper three TSGs. *N* = 3 for all groups. One‐way ANOVA. For *Mbd4*, TAT vs. iBPP, *p = .0434; TAT vs. OE+iBPP, *p = .0221; iBPP vs. HDAC1 OE, ***p = .0005; iBPP vs. OE+iBPP, *p = .0257; HDAC1 OE vs. OE+iBPP, *p = .0372. For *Topors*, TAT vs. iBPP, **p = .001; TAT vs. HDAC1 OE, *p = .0424; iBPP vs. HDAC1 OE, ****p < .0001; iBPP vs. HDAC1 OE, *p = .0143; HDAC1 OE vs. OE+iBPP, **p = .0024. For *Sh2b3*, TAT vs. iBPP, ***p = .0006; TAT vs. HDAC1 OE, **p = .0062; iBPP vs. HDAC1 OE, ****p < .0001; iBPP vs. OE+iBPP, ***p = .0002; HDAC1 OE vs. OE+iBPP, *p = .032. Different letters above columns in the statistical graphs indicate significant difference.

Finally, we verified the dual‐rescue effect of iBPP in mice allografted with mouse ID8 cells, which offers a better reference for future clinical applications than nude mice or BIN2‐KO B6 mice. We also included pyroxamide, an HDAC1 inhibitor,^10^ as a comparison treatment for iBPP. We found that although both HDAC1i and iBPP significantly decreased OC growth (Figure 4A and B), shown as decreased p‐HDAC1 and increased H3K27ac in ovaries (Figure 4C–E) and decreased CD31 and HE4 (Figure S8D–F), only iBPP recovered the number of follicles at each stage (Figure 4F–J) by downregulating p‐RPS6 and upregulating NNT (Figure 4K–M).

**FIGURE 4:**
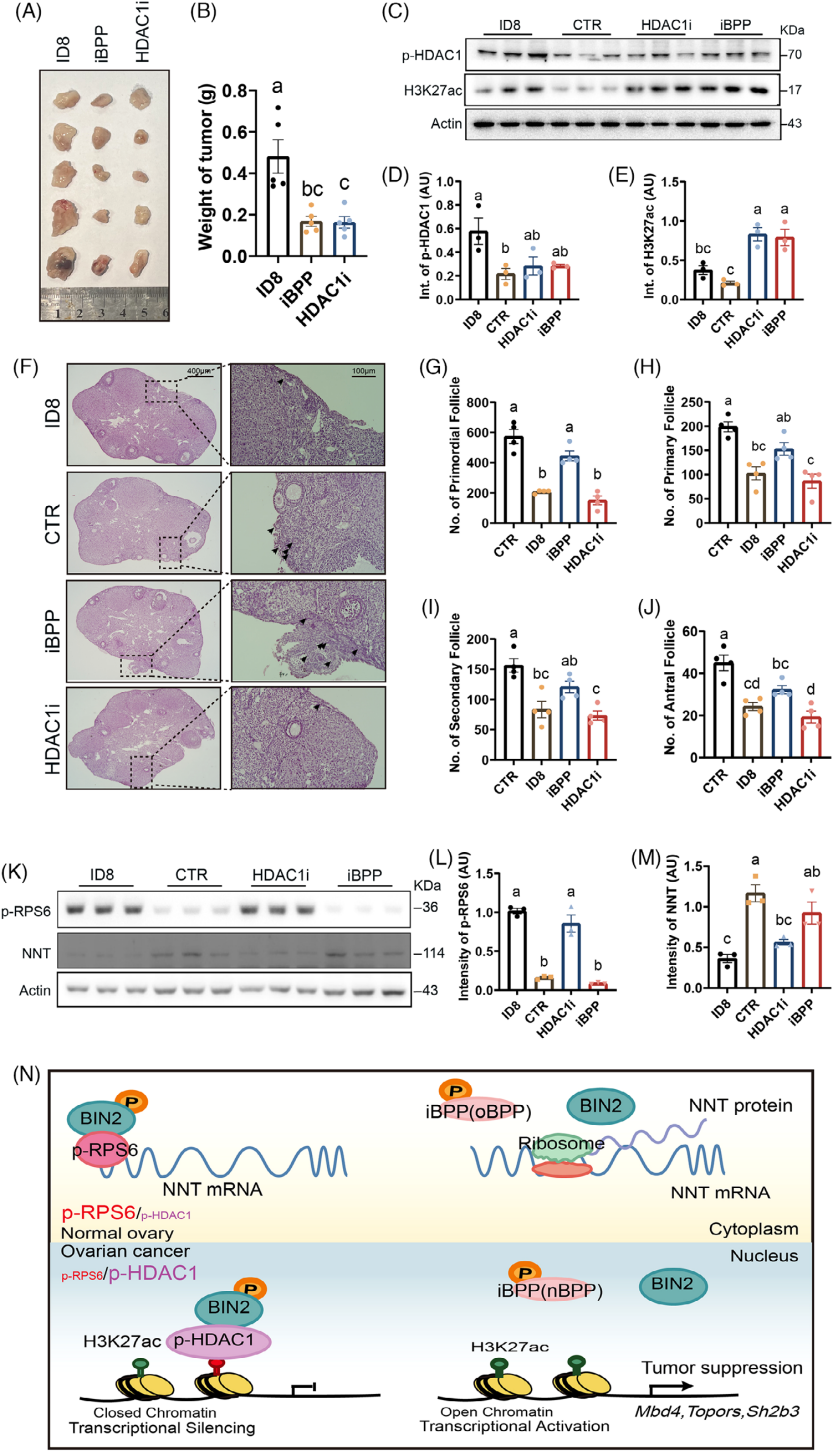
BIN2 inhibition protected ovarian function whereas HDAC1 inhibition didn't in mice under ectopic OC invasion. (A, B) Tumour size measurement showed that both iBPP and HDAC1 inhibitor (HDAC1i) treatment significantly decreased ID8 OC growth. *N* = 5 for all groups. One‐way ANOVA. ID8 vs. iBPP, **p = .0025; ID8 vs. HDAC1i, **p = .0023. (C–E) Blot and quantification showed that both iBPP and HDAC1i treatment significantly reduced p‐HDAC1 and increased H3K27ac in ovaries under ectopic ID8 OC invasion. *N* = 3 for all groups. One‐way ANOVA. For p‐HDAC1, ID8 vs. CTR, *p = .0306. For H3K27ac, ID8 vs. HDAC1i, *p = .0103; ID8 vs. iBPP, *p = .0169; CTR vs. HDAC1i, **p = .0015; CTR vs. iBPP, **p = .0024. (F–J) HE staining of ovarian sections showed that iBPP treatment significantly recovered the numbers of follicles at each stage that were significantly reduced by ectopic ID8 OC invasion; however, HDAC1i treatment had no rescue impact at all. Scale bars for the left row of F, 400 µm. Scale bars for the right row of F (magnified image for the dot‐line region in the left row), 100 µm. Primordial follicles are arrow‐pointed in the right row. *N* = 4 for all groups. One‐way ANOVA. For primordial follicle, CTR vs. ID8, ****p < .0001; CTR vs. HDAC1i, ****p < .0001; ID8 vs. iBPP, **p = .0011; iBPP vs. HDAC1i, ***p = .0002. For primary follicle, CTR vs. ID8, **p = .001; CTR vs. HDAC1i, ***p = .0003; iBPP vs. HDAC1i, *p = .0159. For secondary follicle, CTR vs. ID8, **p = .0021; CTR vs. HDAC1i, ***p = .0007; iBPP vs. HDAC1i, *p = .0396. For antral follicle, CTR vs. ID8, ***p = .0008; CTR vs. iBPP, *p = .0276; CTR vs. HDAC1i, ***p = .0001; iBPP vs. HDAC1i, *p = .0247. (K–M) Blot and quantification showed that iBPP treatment but not HDAC1i treatment significantly recovered ovarian p‐RPS6 and NNT level that were significantly increased (p‐RPS6) or reduced (NNT) by ectopic ID8 OC invasion. *N* = 3 for all groups. One‐way ANOVA. For p‐RPS6, ID8 vs. CTR, ****p < .0001; ID8 vs. iBPP, ****p < .0001; CTR vs. HDAC1i, ***p = .0001; HDAC1i vs. iBPP, ****p < .0001. For NNT, ID8 vs. CTR, **p = .0011; ID8 vs. iBPP, *p = .0103; CTR vs. HDAC1i, **p = .0067. (N) Model: BIN2 Inhibition suppress cancer progression through downregulating p‐HDAC1 in OC, while protect ovarian function through downregulating p‐RPS6. Different letters above columns in the statistical graphs indicate significant difference.

Conclusively, we showed that BIN2 targets HDAC1 and RPS6 within OC and healthy ovaries, respectively. BIN2 inhibition suppresses OC progression by downregulating p‐HDAC1 and upregulating H3K27ac in OC tissue, meanwhile protects the function of healthy ovaries by downregulating p‐RPS6 and upregulating NNT (Figures 4N and S9). This study provides novel therapeutic strategy for young OC patients.

## AUTHOR CONTRIBUTIONS

**Dong Zhang**: Writing—review & editing; writing—original draft; visualisation; validation; supervision; project administration; methodology; investigation; funding acquisition; formal analysis; data curation; conceptualisation. **Cui‐Lian Zhang**: Writing—review & editing; visualisation; validation; supervision; methodology; investigation; funding acquisition; data curation; conceptualisation. **Cong‐Rong Li**: Writing—original draft; project administration; methodology; investigation; formal analysis; data curation; conceptualisation. **Shi‐Ya Xie**: Writing—review & editing; methodology; investigation; formal analysis. **Shu‐Ping Zhang**: Writing—review & editing; methodology; investigation; formal analysis. **Yan‐Jie Yang**: Writing—review & editing; methodology; investigation; formal analysis. **Li‐Li Yang**: Methodology; investigation; formal analysis. **Ying Cao**: Methodology; investigation; formal analysis. **Xiao‐Yan Ying**: Conceptualisation; funding acquisition; resources. **Li‐Li Wang**: Formal analysis; investigation; project administration. **Feng‐Yu Zhu**: Formal analysis; project administration. **Ruo‐Lei Wang**: Formal analysis; project administration. **Zhi‐Xia Yang**: Conceptualisation; funding acquisition; resources. **Chen‐Chen Cui**: Methodology; resources. **Yan‐Ru Li**: Formal analysis; project administration. **Jia‐Ning Xu**: Funding acquisition; resources. **Feng Yue**: Funding acquisition; resources. **Pei‐Zhe Tian**: Software; resources. **Qian Wang**: Funding acquisition; software; resources. **Shao‐Di Zhang**: Funding acquisition; software; resources. **Hong‐Jie Yao**: Funding acquisition; resources. **Yi‐Chun Guan**: Funding acquisition; data curation; conceptualisation.

## CONFLICT OF INTEREST STATEMENT

The authors declared that they have no conflicts of interest to this work.

## FUNDING INFORMATION

This work was financially supported by grants from Major Projects in Provincial and National Union Construction of Henan Medical Science Research Plan (Grant No: SBGJ202001002) to Cui‐Lian Zhang, the General Program of the National Natural Science Foundation of China to Dong Zhang (Grant No: 32070840), the National Key R&D Program of China to Hong‐Jie Yao (Grant No: 2021YFA1100300), the National Key Research & Development Project (Grant No: 2021YFC2700602) to Yi‐Chun Guan, Jiangsu Provincial Key Department of Maternal and Child Health Fund (Grant No: FXK201712) to Xiao‐Yan Ying.

## ETHICS STATEMENT

The collection of OC tissues or normal ovarian tissues from clinical donors was conducted in accordance with relevant guidelines and approved by the Ethical Committee (approval no. [2023]‐KY‐007‐01) of the Second Affiliated Hospital of Nanjing Medical University.

## Supporting information



Supporting Information

Supporting Information

Supporting Information

**Figure S1. Both BIN2 and p‐BIN2 are upregulated in ovarian tumour**. (A–C) Blot and quantification showed that both BIN2 (A and B) and p‐BIN2 (A and C) levels were significantly higher in OC cells (A2780 cells) than in normal ovarian epithelial cells (Moody cells). *N* = 3 for both groups; unpaired two‐tailed *t*‐test. For BIN2, **p = .0021. For p‐BIN2, *p = .0187. (D, E) Immunohistochemistry on a human OC chip showed that p‐BIN2 level was significantly upregulated in high‐grade serious ovarian carcinoma (HGSOC), ovarian endometrioid carcinoma (OEC), and ovarian clear cell carcinoma (CCOC) when compared with that in normal ovaries. Scale bars in D, 200 µm; scale bars in zoom, 50 µm. *N* = 7 for normal ovary and HGSOC groups, *n* = 4 for OEC and OCCC groups. One‐way ANOVA. Normal ovary vs. HGSOC, ****p < .0001. Normal ovary vs. OEC, ***p = .0001. Normal ovary vs. OCCC, ****p < .0001.

**Figure S2. BIN2 knockout recovers the levels of CD31 or Ki67 close to control in in situ chemocarcinogen‐induced ovarian cancer**. (A, B) Immunofluorescence and quantification showed that the number of blood vessel marker CD31 positive cells significantly increased in WT‐Chemo group but much lower in KO‐Chem group. Scale bars, 50 µm. *N* = 16 for all groups. One‐way ANOVA. WT‐CTR vs. WT‐Chem, ***p = .0002; WT‐Chem vs. KO‐Chem, ***p = .0001. (C, D) Immunofluorescence and quantification showed that the number of proliferation marker Ki67 positive cells significantly increased in WT‐Chem group but much lower in KO‐Chem group. Scale bars, 50 µm. *N* = 24 for all groups. One‐way ANOVA. WT‐CTR vs. WT‐Chem, ****p < .0001; WT‐Chem vs. KO‐Chem, **p = .0045.

**Figure S3. BIN2 inhibition impedes A2780 cell growth**. (A) An improved BPP (iBPP), a mixture of omnipresent BPP (oBPP) and nucleus‐targeting BPP (nBPP) was designed to improve the potency to inhibit BIN2 phosphorylation. (B, C) iBPP treatment significantly decreased colony formation of A2780 OC cells. *N* = 3 for both groups; unpaired two‐tailed *t*‐test; **p = .0019. (D, E) Transwell invasion experiment showed that iBPP treatment significantly decreased A2780 OC cell invasion. *N* = 8 for both groups; unpaired two‐tailed *t*‐test; ****p < .0001. (F) Cck8 cell proliferation experiment showed that iBPP treatment significantly decreased A2780 OC cell proliferation. *N* = 7 for both groups; unpaired two‐tailed *t*‐test; **p = .0014. (G, H) Wound healing test showed that iBPP treatment significantly decreased A2780 OC cell migration. *N* = 6 for both groups. Unpaired two‐tailed *t*‐test; for 24 h, ***p < .0005, for 48 h, ****p < .0001. (I–K) PI staining showed that iBPP treatment significantly increased apoptosis level in A2780 OC cells. Scale bars, 20 µm. *N* = 15 for both groups; unpaired two‐tailed *t*‐test; ****p < .0001. (L, M) iBPP treatment significantly decreased mitochondrial membrane potential (J‐aggregate / monomer) in A2780 OC cells. Scale bars, 20 µm. *N* = 14 for both groups; unpaired two‐tailed *t*‐test; ****p < .0001. (N, O) iBPP treatment significantly increased ROS level in A2780 OC cells. Scale bars, 20 µm. *N* = 9 for TAT group, *n* = 25 for iBPP group; unpaired two‐tailed *t*‐test; ****p < .0001.

**Figure S4. BIN2 inhibition impedes the proliferation and migration of HO8910 OC cells**. (A, B) Blot and quantification showed that iBPP treatment significantly decreased p‐BIN2 level in HO8910 OC cells. *N* = 3 for both groups; Unpaired two‐tailed *t*‐test; *p = .0166. (C, D) IBPP treatment significantly decreased colony formation of HO8910 OC cells. *N* = 3 for both groups; unpaired two‐tailed *t*‐test; *p = .0494. (E, F) Transwell invasion experiment showed that iBPP treatment significantly decreased HO8910 OC cell invasion. *N* = 8 for both groups; unpaired two‐tailed *t*‐test; ****p < .0001. (G) Cck8 cell proliferation experiment showed that iBPP treatment significantly decreased HO8910 OC cell proliferation. *N* = 9 for both groups; Unpaired two‐tailed *t*‐test; ****p < .0001. (H, I) Wound healing test showed that iBPP treatment significantly decreased HO8910 OC cell migration. *N* = 6 for both groups. Unpaired two‐tailed *t*‐test; ****p < .0001.

**Figure S5. BIN2 inhibition did not affect the proliferation and migration of Moody cells**. (A, B) Blot and quantification showed that iBPP treatment significantly decreased p‐BIN2 level in Moody cells (normal ovarian epithelial cells). *N* = 3 for both groups; unpaired two‐tailed *t*‐test. *p = .0318. (C, D) IBPP treatment did not affect the colony formation of Moody cells. *N* = 3 for both groups; unpaired two‐tailed *t*‐test. (E, F) Transwell invasion experiment showed that iBPP treatment did not affect the invasion capacity of Moody cells. *N* = 5 for both groups; unpaired two‐tailed *t*‐test. (G) Cck8 cell proliferation experiment showed that iBPP treatment did not affect the proliferation of Moody cells. *N* = 9 for both groups; unpaired two‐tailed *t*‐test. (H, I) Wound healing test showed that iBPP treatment did not affect the migration of Moody cells. *N* = 6 for both groups. Unpaired two‐tailed *t*‐test.

**Figure S6. BIN2 inhibition impedes OC cell lung‐ward metastasis in vivo**. (A, B) iBPP treatment significantly decreased the lung‐ward metastasis (black dot‐line encircled) area of ID8 mouse OC cell. *N* = 6 for all groups; Unpaired two‐tailed *t*‐test. ***p = .0003. (C) HE staining of mice lung tissue containing metastasis show that ID8 lung‐ward metastasis significantly altered lung structure, whereas iBPP treatment partially recovered lung structure. Scale bars, 200 µm. (D, E) Immunohistochemical staining showed that ID8 lung‐ward metastasis significantly increased CD31 level, whereas iBPP treatment tended to recovered CD31 level. Scale bars, 50 µm. *N* = 9 for all groups. One‐way ANOVA. PBS vs. ID8+TAT (control peptide), ***p = .0009.

**Figure S7. BIN2 inhibition impede OC progression through upregulating H3K27ac**. (A, B) Blot and quantification showed that iBPP treatment did not increase the level of H3K9ac and H3K14ac in A2780 OC cells. *N* = 3 for both groups; unpaired two‐tailed *t*‐test. **p = .0095. (C–E) KEGG analysis CUT & TAG Seq showed that after iBPP treatment, H3K27ac‐binding gene‐region peaks were more cancer‐related (red dot‐line rectangle) than H3K9ac‐ or H3K14ac‐binding peaks. (F, G) Reverse Transcription PCR and quantification showed that iBPP treatment did not affect the expression of some key tumour suppressor genes (*Brca1*, *Brca2*, *Tp53*) and oncogenes (*Pik3ca*, *Kras*) implicated in ovarian oncogenesis in A2780 OC cells. *N* = 3 for both groups; unpaired two‐tailed *t*‐test.

**Figure S8. BIN2 Inhibition and HDAC1 inhibition had similar impacts on ectopic ID8 OC tissues**. (A–C) Blot and quantification showed that both iBPP and HDAC1i treatment significantly decreased p‐HDAC1 while increased H3K27ac in ectopic ID8 OC tissues. *N* = 3 for all groups. One‐way ANOVA. For p‐HDAC1, ID8 vs. iBPP, **p = .0098; ID8 vs. HDAC1i, *p = .0116. For H3K27ac, ID8 vs. iBPP, *p = .0361; ID8 vs. HDAC1i, **p = .0013; iBPP vs. HDAC1i, *p = .0361. (D–F) Blot and quantification showed that both iBPP and HDAC1i treatment significantly decreased tumour markers CD31 and HE4 in ectopic ID8 OC tissues. *N* = 3 for all groups. One‐way ANOVA. For CD31, ID8 vs. iBPP, **p = .0098; ID8 vs. HDAC1i, **p = .0043. For HE4, ID8 vs. iBPP, *p = .02; ID8 vs. HDAC1i, *p = .046.

**Figure S9. Model: BIN2 Inhibition suppress cancer progression through downregulating p‐HDAC1 in OC tissues, while protect ovarian function through downregulating p‐RPS6 in healthy ovarian tissues**. In OC tissues, p‐HDAC1 dominates over p‐RPS6 in binding p‐BIN2, while p‐HDAC1 negatively regulates H3K27ac; therefore, BIN2 inhibition by iBPP upregulates tumour suppressor genes through downregulating p‐HDAC1 and upregulating H3K27ac. In the remaining healthy ovarian tissues, p‐RPS6 dominates over p‐HDAC1 in binding p‐BIN2; therefore, BIN2 inhibition by iBPP increases the numbers of follicles at each stage and improves oocyte quality through downregulating p‐RPS6 and upregulating NNT.

Supporting Information

Supporting Information
