# Supplementary figures and images for: BIN2 inhibition suppress ovarian cancer progression meanwhile protect ovarian function through downregulating HDAC1 and RPS6 phosphorylation respectively
Source: Clin Transl Med. 2024 Oct 16;14(10):e70051. doi: 10.1002/ctm2.70051 (PMC11480968; doi:10.1002/ctm2.70051)

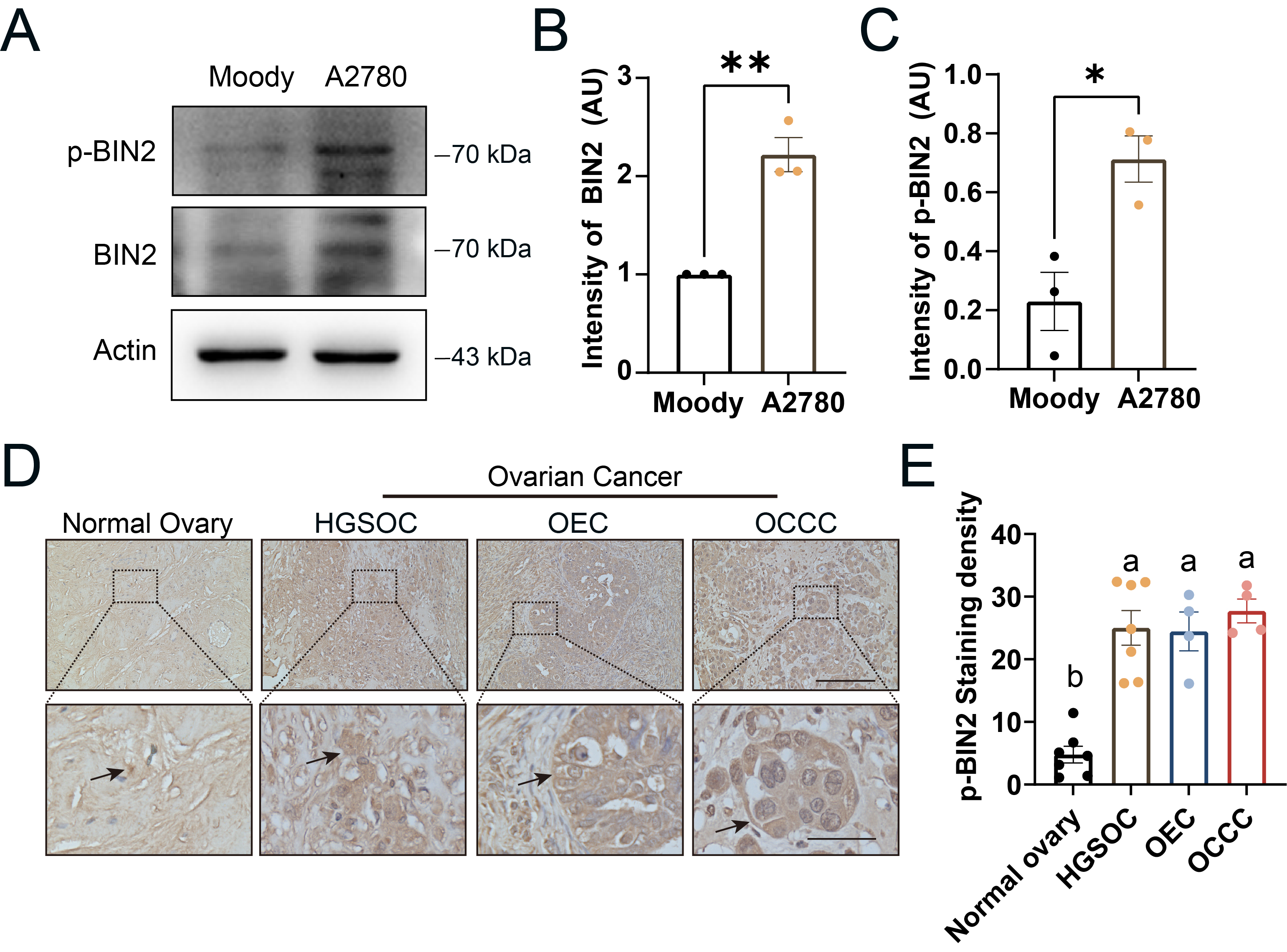

Supplement: Supplementary file 4 — Figure S1. Both BIN2 and p‐BIN2 are upregulated in ovarian tumour. (A–C) Blot and quantification showed that both BIN2 (A and B) and p‐BIN2 (A and C) levels were significantly higher in OC cells (A2780 cells) than in normal ovarian epithelial cells (Moody cells). N = 3 for both groups; unpaired two‐tailed t‐test. For BIN2, **p = .0021. For p‐BIN2, *p = .0187. (D, E) Immunohistochemistry on a human OC chip showed that p‐BIN2 level was significantly upregulated in high‐grade serious ovarian carcinoma (HGSOC), ovarian endometrioid carcinoma (OEC), and ovarian clear cell carcinoma (CCOC) when compared with that in normal ovaries. Scale bars in D, 200 µm; scale bars in zoom, 50 µm. N = 7 for normal ovary and HGSOC groups, n = 4 for OEC and OCCC groups. One‐way ANOVA. Normal ovary vs. HGSOC, ****p < .0001. Normal ovary vs. OEC, ***p = .0001. Normal ovary vs. OCCC, ****p < .0001. [file CTM2-14-e70051-s001.jpg]

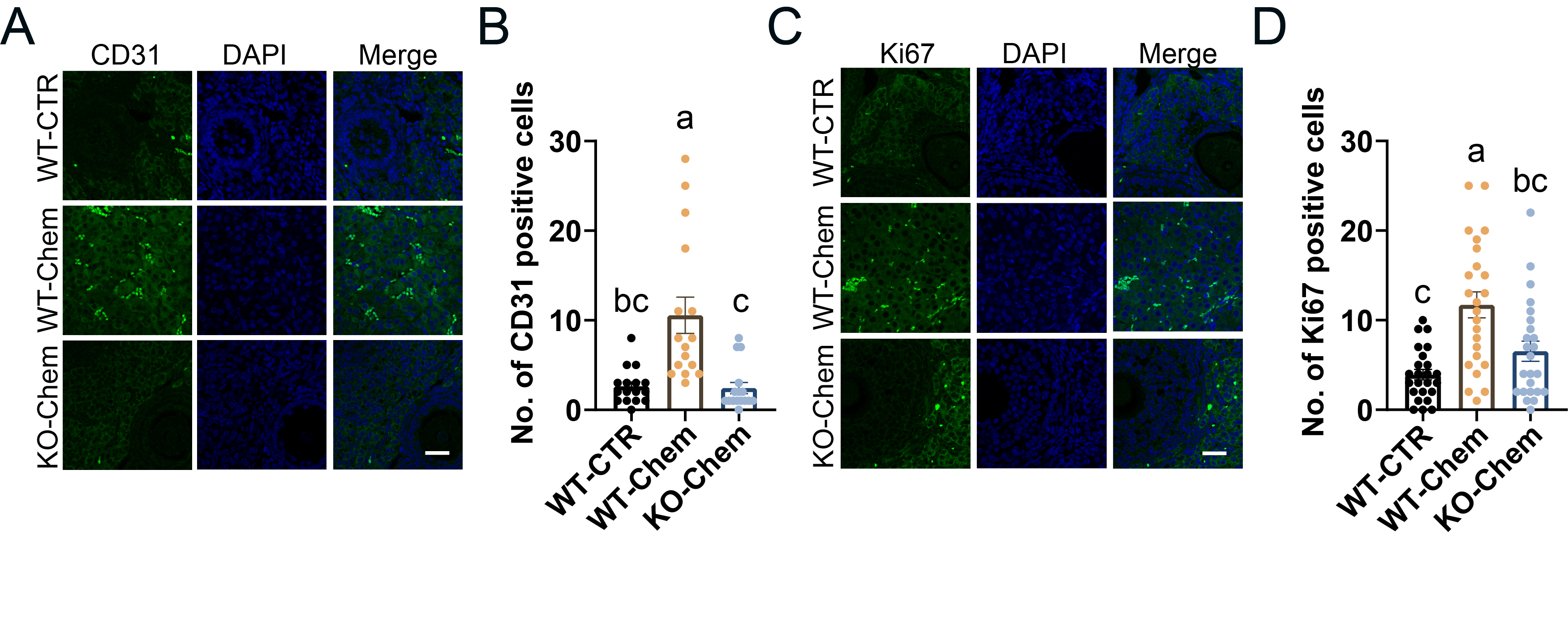

Supplement: Supplementary file 5 — Figure S2. BIN2 knockout recovers the levels of CD31 or Ki67 close to control in in situ chemocarcinogen‐induced ovarian cancer. (A, B) Immunofluorescence and quantification showed that the number of blood vessel marker CD31 positive cells significantly increased in WT‐Chemo group but much lower in KO‐Chem group. Scale bars, 50 µm. N = 16 for all groups. One‐way ANOVA. WT‐CTR vs. WT‐Chem, ***p = .0002; WT‐Chem vs. KO‐Chem, ***p = .0001. (C, D) Immunofluorescence and quantification showed that the number of proliferation marker Ki67 positive cells significantly increased in WT‐Chem group but much lower in KO‐Chem group. Scale bars, 50 µm. N = 24 for all groups. One‐way ANOVA. WT‐CTR vs. WT‐Chem, ****p < .0001; WT‐Chem vs. KO‐Chem, **p = .0045. [file CTM2-14-e70051-s012.jpg]

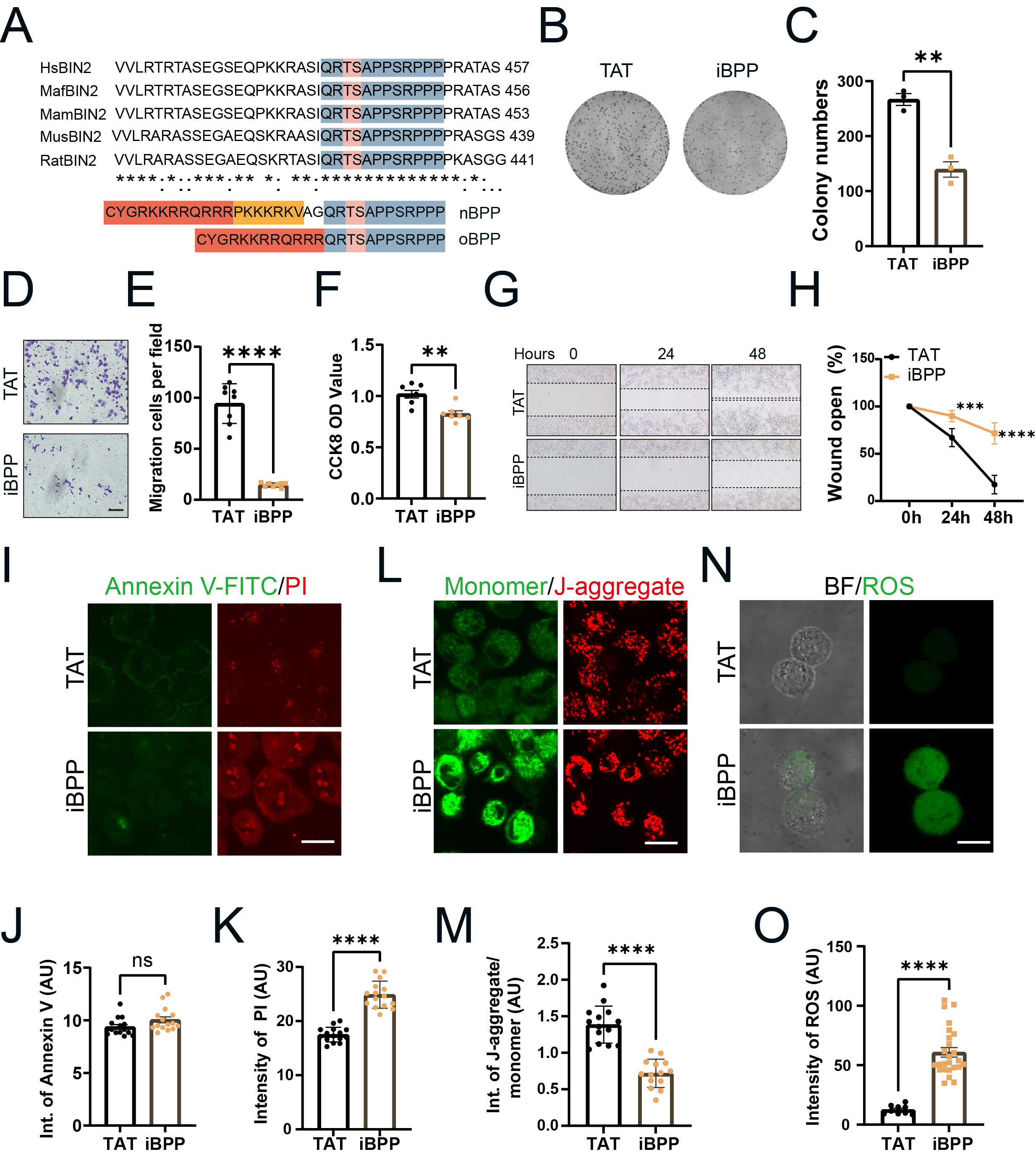

Supplement: Supplementary file 6 — Figure S3. BIN2 inhibition impedes A2780 cell growth. (A) An improved BPP (iBPP), a mixture of omnipresent BPP (oBPP) and nucleus‐targeting BPP (nBPP) was designed to improve the potency to inhibit BIN2 phosphorylation. (B, C) iBPP treatment significantly decreased colony formation of A2780 OC cells. N = 3 for both groups; unpaired two‐tailed t‐test; **p = .0019. (D, E) Transwell invasion experiment showed that iBPP treatment significantly decreased A2780 OC cell invasion. N = 8 for both groups; unpaired two‐tailed t‐test; ****p < .0001. (F) Cck8 cell proliferation experiment showed that iBPP treatment significantly decreased A2780 OC cell proliferation. N = 7 for both groups; unpaired two‐tailed t‐test; **p = .0014. (G, H) Wound healing test showed that iBPP treatment significantly decreased A2780 OC cell migration. N = 6 for both groups. Unpaired two‐tailed t‐test; for 24 h, ***p < .0005, for 48 h, ****p < .0001. (I–K) PI staining showed that iBPP treatment significantly increased apoptosis level in A2780 OC cells. Scale bars, 20 µm. N = 15 for both groups; unpaired two‐tailed t‐test; ****p < .0001. (L, M) iBPP treatment significantly decreased mitochondrial membrane potential (J‐aggregate / monomer) in A2780 OC cells. Scale bars, 20 µm. N = 14 for both groups; unpaired two‐tailed t‐test; ****p < .0001. (N, O) iBPP treatment significantly increased ROS level in A2780 OC cells. Scale bars, 20 µm. N = 9 for TAT group, n = 25 for iBPP group; unpaired two‐tailed t‐test; ****p < .0001. [file CTM2-14-e70051-s008.jpg]

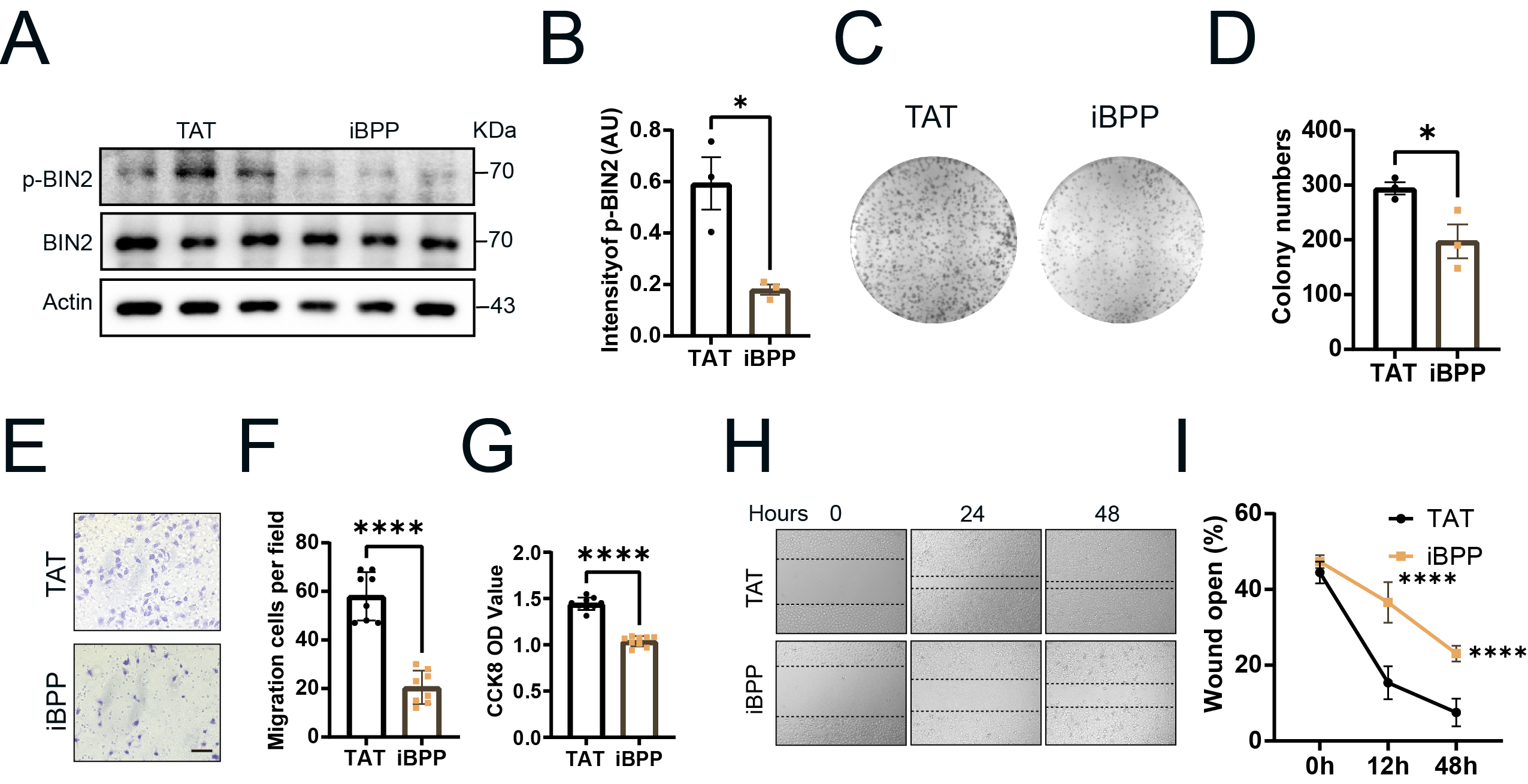

Supplement: Supplementary file 7 — Figure S4. BIN2 inhibition impedes the proliferation and migration of HO8910 OC cells. (A, B) Blot and quantification showed that iBPP treatment significantly decreased p‐BIN2 level in HO8910 OC cells. N = 3 for both groups; Unpaired two‐tailed t‐test; *p = .0166. (C, D) IBPP treatment significantly decreased colony formation of HO8910 OC cells. N = 3 for both groups; unpaired two‐tailed t‐test; *p = .0494. (E, F) Transwell invasion experiment showed that iBPP treatment significantly decreased HO8910 OC cell invasion. N = 8 for both groups; unpaired two‐tailed t‐test; ****p < .0001. (G) Cck8 cell proliferation experiment showed that iBPP treatment significantly decreased HO8910 OC cell proliferation. N = 9 for both groups; Unpaired two‐tailed t‐test; ****p < .0001. (H, I) Wound healing test showed that iBPP treatment significantly decreased HO8910 OC cell migration. N = 6 for both groups. Unpaired two‐tailed t‐test; ****p < .0001. [file CTM2-14-e70051-s009.jpg]

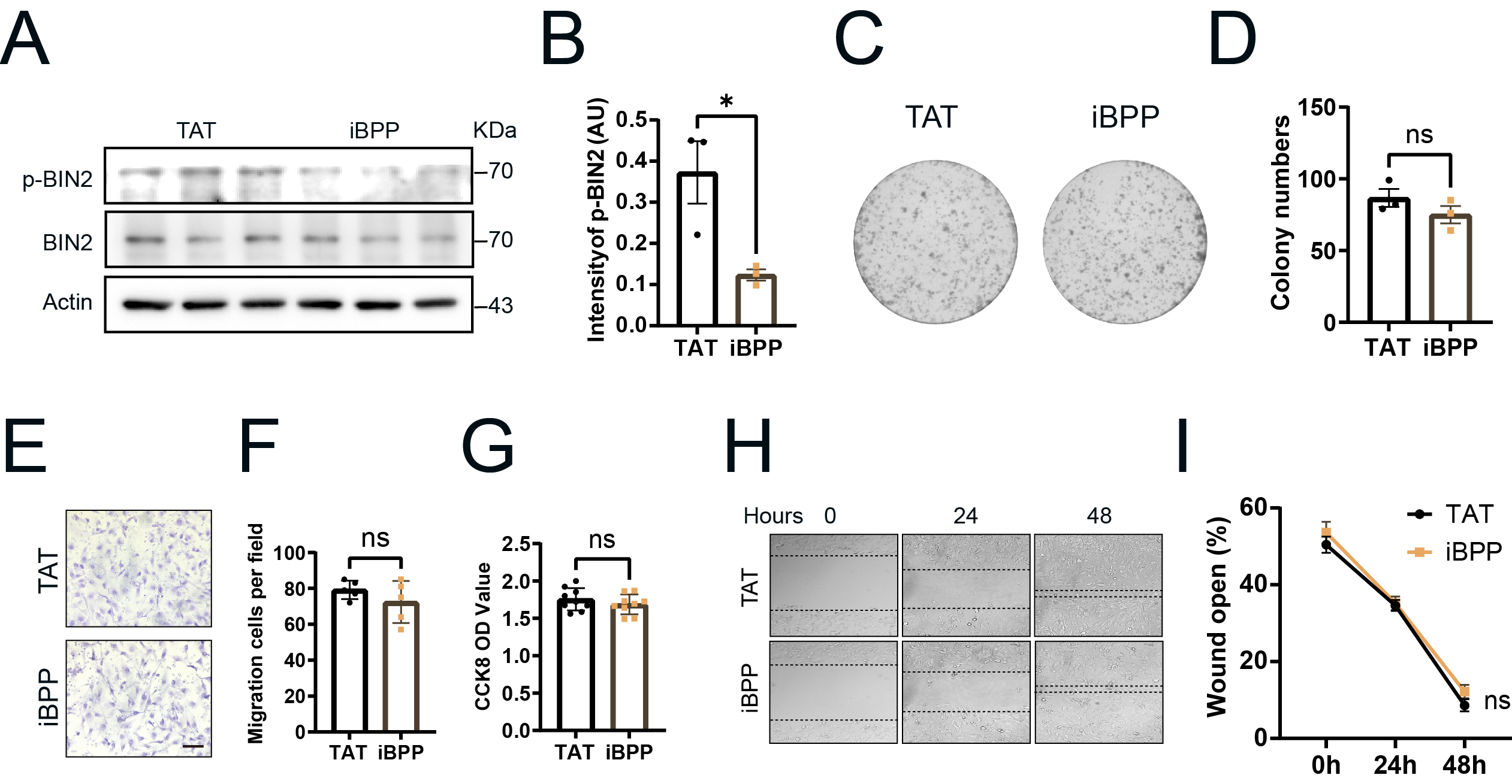

Supplement: Supplementary file 8 — Figure S5. BIN2 inhibition did not affect the proliferation and migration of Moody cells. (A, B) Blot and quantification showed that iBPP treatment significantly decreased p‐BIN2 level in Moody cells (normal ovarian epithelial cells). N = 3 for both groups; unpaired two‐tailed t‐test. *p = .0318. (C, D) IBPP treatment did not affect the colony formation of Moody cells. N = 3 for both groups; unpaired two‐tailed t‐test. (E, F) Transwell invasion experiment showed that iBPP treatment did not affect the invasion capacity of Moody cells. N = 5 for both groups; unpaired two‐tailed t‐test. (G) Cck8 cell proliferation experiment showed that iBPP treatment did not affect the proliferation of Moody cells. N = 9 for both groups; unpaired two‐tailed t‐test. (H, I) Wound healing test showed that iBPP treatment did not affect the migration of Moody cells. N = 6 for both groups. Unpaired two‐tailed t‐test. [file CTM2-14-e70051-s007.jpg]

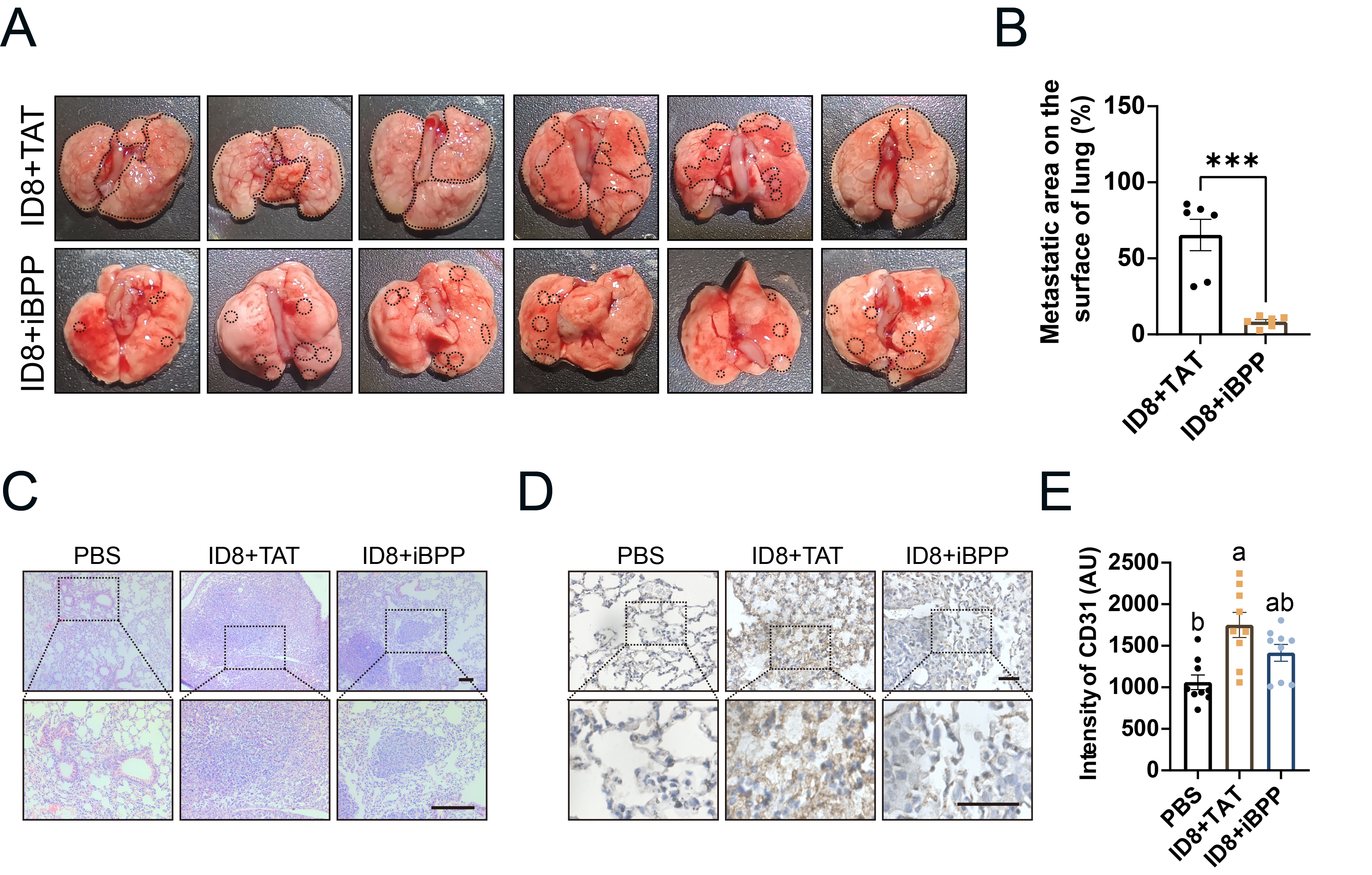

Supplement: Supplementary file 9 — Figure S6. BIN2 inhibition impedes OC cell lung‐ward metastasis in vivo. (A, B) iBPP treatment significantly decreased the lung‐ward metastasis (black dot‐line encircled) area of ID8 mouse OC cell. N = 6 for all groups; Unpaired two‐tailed t‐test. ***p = .0003. (C) HE staining of mice lung tissue containing metastasis show that ID8 lung‐ward metastasis significantly altered lung structure, whereas iBPP treatment partially recovered lung structure. Scale bars, 200 µm. (D, E) Immunohistochemical staining showed that ID8 lung‐ward metastasis significantly increased CD31 level, whereas iBPP treatment tended to recovered CD31 level. Scale bars, 50 µm. N = 9 for all groups. One‐way ANOVA. PBS vs. ID8+TAT (control peptide), ***p = .0009. [file CTM2-14-e70051-s013.jpg]

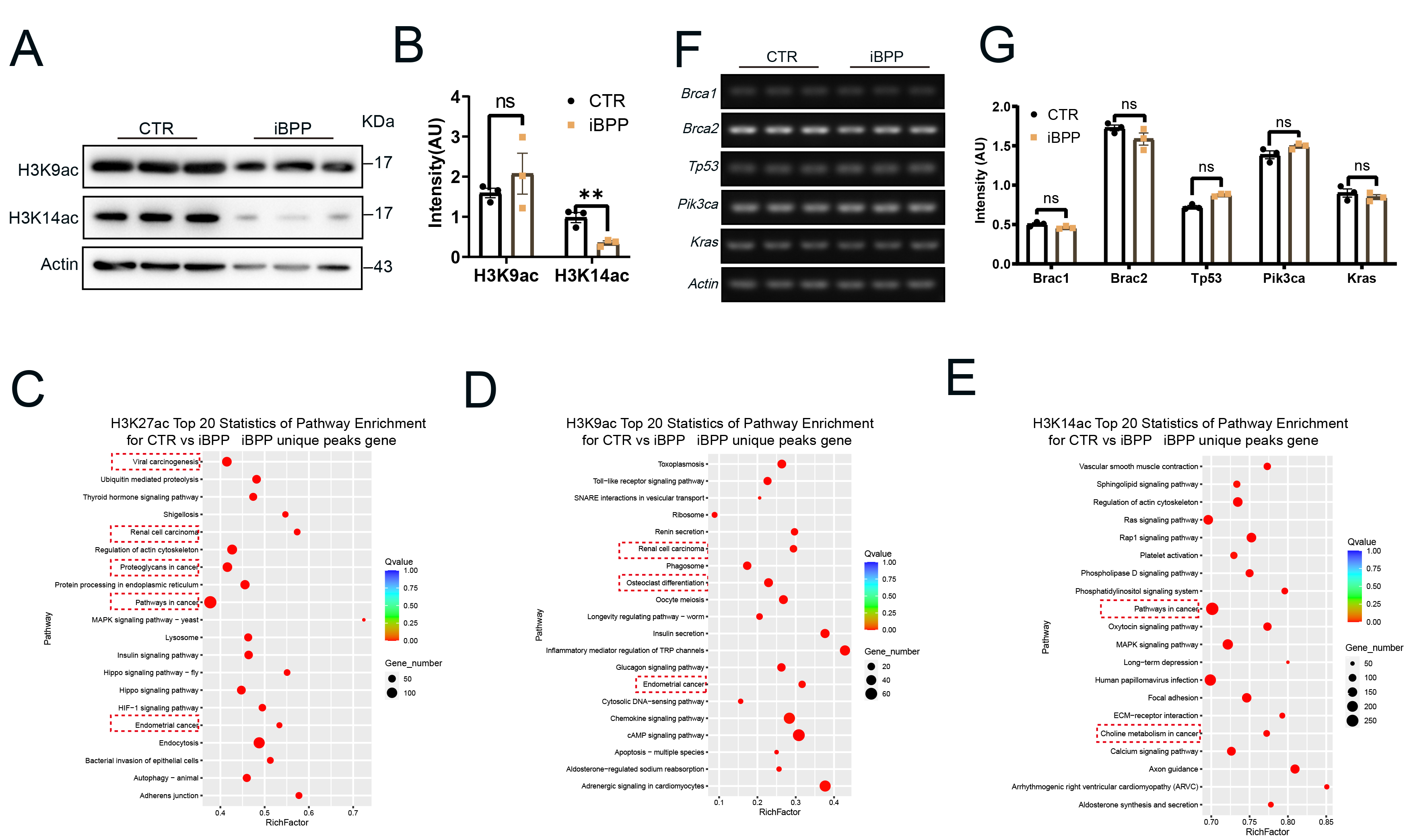

Supplement: Supplementary file 10 — Figure S7. BIN2 inhibition impede OC progression through upregulating H3K27ac. (A, B) Blot and quantification showed that iBPP treatment did not increase the level of H3K9ac and H3K14ac in A2780 OC cells. N = 3 for both groups; unpaired two‐tailed t‐test. **p = .0095. (C–E) KEGG analysis CUT & TAG Seq showed that after iBPP treatment, H3K27ac‐binding gene‐region peaks were more cancer‐related (red dot‐line rectangle) than H3K9ac‐ or H3K14ac‐binding peaks. (F, G) Reverse Transcription PCR and quantification showed that iBPP treatment did not affect the expression of some key tumour suppressor genes (Brca1, Brca2, Tp53) and oncogenes (Pik3ca, Kras) implicated in ovarian oncogenesis in A2780 OC cells. N = 3 for both groups; unpaired two‐tailed t‐test. [file CTM2-14-e70051-s004.jpg]

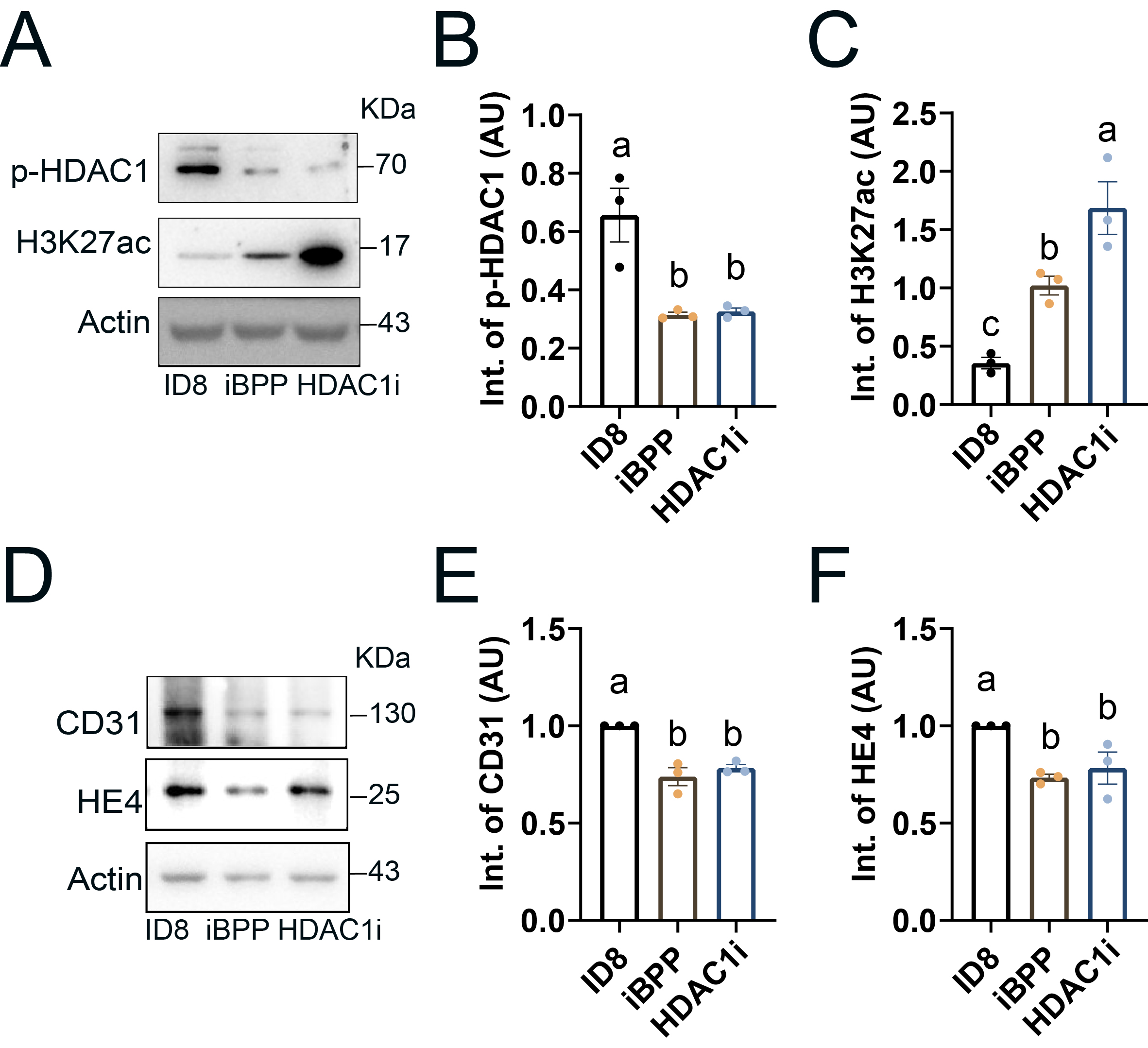

Supplement: Supplementary file 11 — Figure S8. BIN2 Inhibition and HDAC1 inhibition had similar impacts on ectopic ID8 OC tissues. (A–C) Blot and quantification showed that both iBPP and HDAC1i treatment significantly decreased p‐HDAC1 while increased H3K27ac in ectopic ID8 OC tissues. N = 3 for all groups. One‐way ANOVA. For p‐HDAC1, ID8 vs. iBPP, **p = .0098; ID8 vs. HDAC1i, *p = .0116. For H3K27ac, ID8 vs. iBPP, *p = .0361; ID8 vs. HDAC1i, **p = .0013; iBPP vs. HDAC1i, *p = .0361. (D–F) Blot and quantification showed that both iBPP and HDAC1i treatment significantly decreased tumour markers CD31 and HE4 in ectopic ID8 OC tissues. N = 3 for all groups. One‐way ANOVA. For CD31, ID8 vs. iBPP, **p = .0098; ID8 vs. HDAC1i, **p = .0043. For HE4, ID8 vs. iBPP, *p = .02; ID8 vs. HDAC1i, *p = .046. [file CTM2-14-e70051-s014.jpg]

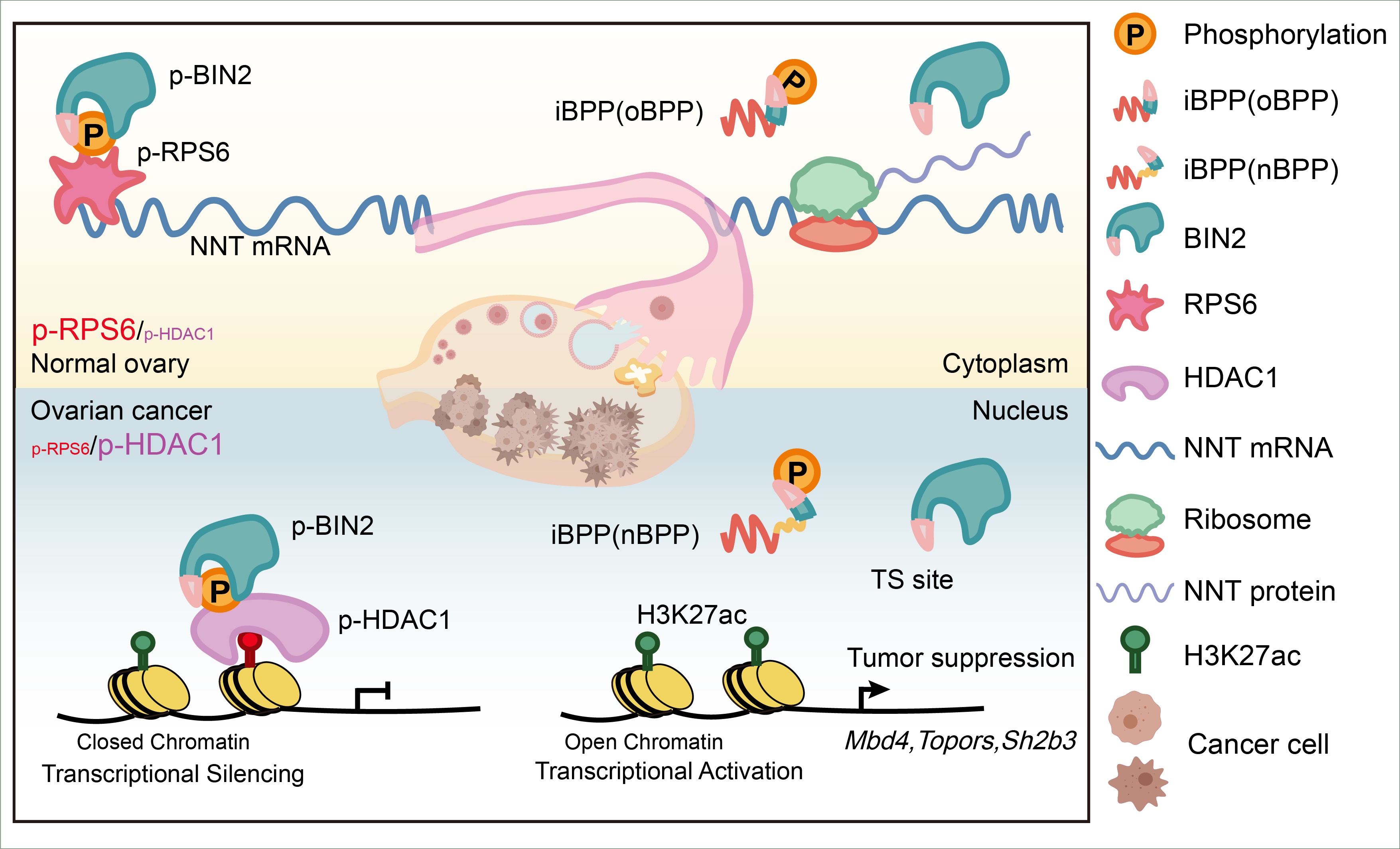

Supplement: Supplementary file 12 — Figure S9. Model: BIN2 Inhibition suppress cancer progression through downregulating p‐HDAC1 in OC tissues, while protect ovarian function through downregulating p‐RPS6 in healthy ovarian tissues. In OC tissues, p‐HDAC1 dominates over p‐RPS6 in binding p‐BIN2, while p‐HDAC1 negatively regulates H3K27ac; therefore, BIN2 inhibition by iBPP upregulates tumour suppressor genes through downregulating p‐HDAC1 and upregulating H3K27ac. In the remaining healthy ovarian tissues, p‐RPS6 dominates over p‐HDAC1 in binding p‐BIN2; therefore, BIN2 inhibition by iBPP increases the numbers of follicles at each stage and improves oocyte quality through downregulating p‐RPS6 and upregulating NNT. [file CTM2-14-e70051-s006.jpg]
