# Supplementary material for: BIN2 inhibition suppress ovarian cancer progression meanwhile protect ovarian function through downregulating HDAC1 and RPS6 phosphorylation respectively
Source: Clin Transl Med. 2024 Oct 16;14(10):e70051. doi: 10.1002/ctm2.70051 (PMC11480968; doi:10.1002/ctm2.70051)
Supplement: Supplementary file 13 — Supporting Information [file CTM2-14-e70051-s011.doc]

**BIN2 Inhibition Suppress Ovarian Cancer Progression Meanwhile Protect Ovarian function through Downregulating HDAC1 and RPS6 Phosphorylation Respectively**

**Materials and methods**

**Knockout mice**

All animal experiments in this study were approved by the Animal Care and Use Committee (IACUC) of Nanjing Medical University (NJMU) with approval number IACUC-2205056. All mice were raised under standard specific pathogen-free (SPF) conditions in the Animal Core Facility (ACF). Mice were anesthetized with carbon dioxide and then sacrificed by cervical dislocation before the removal of ovarian or other tissue. In our previous Bin2 study, global Bin2-KO C57BL/6 mice were created. Genotyping was also performed as described previously.

In fertility trials, five wild-type or five *Bin2*-knockout female mice were mated with wild-type male mice of 2 months of age (all C57BL/6 mice) starting 1 month after tumor-inducing surgery.

**Model mice**

Three types of tumor model mice were created.

1) Chemical carcinogen-induced tumors in B6 mice

Two chemical carcinogens (chemicarcinogen), namely 4 mg/mL of 7,12-dimethylbenz[a]anthracene (DMBA) and 4 mg/mL of N-methyl-N-nitrosourea (MNU) were used to fully soak a 1 mm × 1 mm filter paper. Then, the filter paper was transplanted into the subcapsular part of the WT or *Bin2*-KO ovary for in situ tumorigenesis induction. The induction lasted for 2 months.

2) Xenograft tumors in immunodeficient mice

Immunodeficient Balb/c-Fox1nuAusb mice (nude mice) were purchased from VitalRiver (Beijing). A2780 OC cells (1 × 10^7^ cells per mouse) were transplanted subcutaneously into the left axilla of 2-month-old female immunodeficient nude mice. The length and width of the tumor were measured with a digital caliper and the tumor size was calculated by the following formula: volume = 0.5 × (width)^2^ × length. The tumor-bearing period was 3 weeks, and the mice were sacrificed by anesthesia after 3 weeks.

3) Allograft tumors in B6 mice

For follicular counting, ID8 OC cells (tumor-forming in normal mice, 1 × 10^7^ cells per mouse) were transplanted subcutaneously into the left axilla of 2-month-old female normal B6 mice. After 4 weeks, the mice were injected with five units of PMSG and sacrificed using anesthesia 24 h later.

**Antibodies**

Information on all commercial primary and secondary antibodies is included in Supplementary Table 1.

A rabbit anti-BIN2-T423^p^S421^p^ polyclonal antibody was raised against QSKRAASIQRT(phospho)S(phospho)A by Zoonbio BioTech and purified by affinity purification; rabbit anti-HDAC2-S424^p^ was raised against SDS(phospho)EDEGEGGRRC by Zoonbio BioTech and purified by affinity purification.

**Injection of competitive peptides in animals**

To inhibit the phosphorylation of cytoplasmic BIN2 at the T423 and S424 sites, the TAT sequence (CYGRKKRRQRRR) was fused with QRTSAPPSRPPP (T423 and S424 underlined), a sequence containing the phosphorylation sites in BIN2, called omnipresent BPP (oBPP).

For the phosphorylation inhibition of nucleic BIN2 at T423 and S424, the nuclear localization sequence (NLS), PKKKRKV, was fused between TAT and QRTSAPPSRPPP, named nucleus-targeting BPP (nBPP). In all experiments, a mixture of oBPP and nBPP was used to enhance the effect of inhibiting BIN2 phosphorylation, and the mixture was named improved BPP (iBPP).

iBPP was dissolved in 10% DMSO (Sigma) diluted from sterile ultra-pure water. The concentration of the concentrated liquid was 5 mg/mL. The concentrated storage solution was diluted with 0.9% sodium chloride solution to a final concentration of 0.1 mg/mL, and a dose of 0.5 mg/kg (for a 25 g mouse, the injection volume was 125 μL) was injected into the abdominal cavity or the tumor. The TAT-only sequence was injected into control mice as a control peptide.

**Intra-tumoral injection of the HDAC1 inhibitor pyroxamide**

Subcutaneous cell line-derived tumor homotransplantation studies were performed by implanting ID8 cells (1 × 10^7^ cells per mouse in 100 μL PBS) into female 8-week-old C57BL/6 mice.

The HDAC1 inhibitor pyroxamide was concentrated with 10% DMSO (Sigma), 40% PEG300, 5% Tween-80, and 45% saline solution. The final concentration was 5 mg/mL, which was diluted to a working concentration of 1 mg/mL with 0.9% sodium chloride solution, and the dose for intra-tumoral injection was 5 mg/kg/day.

**Cell culture, plasmid transfection**

Human EOC cells, A2780, were obtained from ECACC (Procell Life Science & Technology, cat. no. 93112519). Cells were cultured in DMEM with 10% fetal bovine serum (FBS).

Mouse EOC cells, ID8, were obtained from Millipore (EK bioscience, cat. No. CC-Y2121). The cells were cultured in DMEM containing 10% FBS.

During plasmid transfection, cells grew at a density of 60–70%. The pcDNA3.1+ plasmid was transfected with Lipo6000^TM^ transfection reagent and an empty plasmid was used as control.

**Apoptosis detection**

The Annexin V-fluorescein isothiocyanate (FITC)/ PI Apoptosis Detection Kit (Yeasen) was used to detect apoptosis levels. The cells were first cultured on a cover slide and stained in darkness at room temperature for 15 min with 5 μL Annexin V-FITC and 10 μL PI staining solution. After washing with PBS, the cells were photographed with a confocal microscope.

**Assay of mitochondrial transmembrane potential**

A2780 cells were cultured on a cover slide and treated with peptides for 24 h. Then, cells were treated with a 1:200 dilution of fluorescent potential indicator-1 (cat. no. 40706ES60, Yeasen) and incubated at 37°C for 20 min, then washed twice with JC-1 washing solution. Images of green fluorescence (JC-1 as a monomer at low membrane potential) and red fluorescence (JC-1 as an aggregate at high membrane potential) were taken by confocal microscopy. A decrease in the red/green fluorescence intensity ratio indicated mitochondrial depolarization.

**Detection of ROS generation**

A ROS assay kit (cat. no. S0033, Beyotime) was used to detect ROS generation in A2780 cells. In brief, A2780 cells were mounted onto slides and treated with peptides, then incubated with a dichlorofluorescein diacetate probe for 20 min at 37°C in the dark and washed for confocal imaging.

**Cell proliferation assay**

The impact of iBPP on cell proliferation was assessed using Cell Counting Kit-8 (CCK-8, BD0079-1, Bioworld). Cells in the logarithmic growth phase were initially seeded into 96-well plates at a density of 5 × 10^3^ cells per well. Subsequently, 10 μL of CCK-8 solution was added to each well, and samples were incubated for 4 h. The absorbance, which is directly proportional to the number of proliferating cells, was then measured at an optical density (OD) of 450 nm.

**Wound healing assay**

A total of 5 × 10^5^ cells per well were cultured in 6-well plates until they reached 80–90% confluence. The cells were then treated with 40 μg/mL of either the iBPP peptide or the TAT peptide. The cell monolayer was carefully scratched using a sterile 200 μL pipette tip to create a wound. Three pictures of randomly chosen wound areas were captured at designated time points (0, 24, and 48 h) using a light microscope from Olympus Corp in Tokyo, Japan. The wound areas were later analyzed using Image J software.

**Colony formation assay**

A total of 300 cells were distributed into each well of a 6-well plate and subjected to a 7-day culture. During this period, two different peptide treatments were applied: iBPP peptide at a concentration of 40 μg/mL and TAT peptide. Following the culture, the wells were rinsed with PBS, fixed with 1% paraformaldehyde (PFA), and subsequently stained with 0.1% crystal violet. Finally, observations were made using a light microscope from Nikon in Tokyo, Japan.

**Ovarian hematoxylin and eosin (HE) staining and follicle counting**

To eliminate the effects of distinct estrous cycles among different mice, 10 IU / mice PMSG were intraperitoneally injected into mice to synchronize the estrous cycle. Ovaries were obtained after 48 hours, washed, and fixed in 10% buffered formalin or 4% PFA overnight, embedded in paraffin, continuously sectioned at 5 µm thickness (about 240 slices totally for an ovary) and mounted onto coverslips (about three row x 10 slice per coverslip), then stained with hematoxylin and eosin.

Pederson’s standard was used to classify the stages of follicles, with only follicles featuring visible nuclei being included. In short, a primordial or primary follicle was defined as an oocyte surrounded by a single layer of flattened or cuboidal granulosa cells, while a secondary follicle was defined as an oocyte encircled by more than one layer of cuboidal granulosa cells without a visible cavity. An antral follicle, on the other hand, was characterized by the presence of a clear cavity filled with follicular fluid. To determine the follicle count, every other two slices were counted to avoid repetitive inclusion of the same follicle, and the final number of follicles within each stage was a cumulative total of all corresponding counts observed. Ovaries from four mice (n = 4) were used for each group.

**In vitro phosphorylation assays**

In vitro phosphorylation assays were performed using BRB80 solution containing 10% glycerol, 1 mM ATP, and 5 mM DTT. BIN2 proteins and HDAC1 were mixed in the solution in suitable quantities and incubated at room temperature for 20 min. The reaction was then subjected to western blotting for further analysis.

**RNA sequencing and analysis**

RNA samples were collected from the ovaries of mice. Seq Health Technology Co., Ltd. (Wuhan, China) carried out RNA isolation, RNA high-throughput sequencing, and data analysis following standard procedures. The library products were sequenced on a DNBSEQ-T7 sequencer (manufactured by MGI Tech Co., Ltd., in China) using the PE150 model. The significance of the differentially expressed genes (DEGs) in gene expression analysis was determined by the bioinformatics services of Seq Health Technology Co., Ltd., based on the criteria of |log2 (treated/control)| ≥ 1.2 and q value < 0.001. All original sequence datasets have been submitted to the NCBI Sequence Read Archive database under an accession number. For the RNA-seq in Fig. 5I, the accession numbers for CTR1-3 and iBPP1-3 are GSM7831887–GSM7831892 (six continuous numbers).

**CUT&Tag sequencing data processing and analysis**

The experiment utilized the Hyperactive Universal CUT&Tag Assay Kit for Illumina Pro (Vazyme, TD904). Data processing for CUT&Tag sequencing followed established procedures. Specifically, the paired-end reads were aligned to GRCh38p13 using bowtie2 (v2.4.1). Subsequently, the deepTools (v3.3.0) software was employed to generate a BigWig file, which allowed the visualization of dense, continuous data as a graph. The normalization method applied during this process was CPM. The Integrative Genomics Viewer (IGV) was used to visualize the results.

For the CUT&Tag-seq depicted in Fig. 5G, the accession numbers for iBPP, CTR, and IgG are GSM7831884–GSM7831886 (three consecutive numbers).

**Plasmid construction**

High-fidelity DNA polymerase (Vanzyme) was used to amplify the full-length CDS or domain fragment of a gene from cDNA reverse-transcribed by SSRT VI (Thermo Fisher). The resulting PCR products were purified and digested using high-fidelity restriction enzymes (NEB), then inserted into related empty plasmids using Quick ligase (NEB). The primer sequences used for the constructs can be found in Supplementary Table 2. In all constructs, full-length or other fragments were linked to the Strep II or FLAG tag for quick detection with Strep II or FLAG antibodies. Detailed information about the inserts and vectors and the corresponding figure panels utilizing the related constructs can be found in Supplementary Table 3.

**Immunoprecipitation and mass spectrometry**

For immunoprecipitation, BIN2-WT, AA, or DD mutant plasmids were separately introduced into ID8 cells. Following this, 2 × 10^6^ cells were lysed in 250 μL of IP buffer and pre-cleared using protein A/G beads (Yeasen, Beijing, China) for 4 h at 4°C. Next, 2.5 μg of mouse anti-Strep II monoclonal antibody was initially bound to 30 μL protein-A/G beads (Yeasen, Beijing, China) for 4 h at 4°C on a rotating wheel in 250 mL IP buffer containing 20 mM Tris-HCl (pH 8.0), 10 mM EDTA, 1 mM EGTA, 150 mM NaCl, 0.05% Triton X-100, 0.05% NP-40, 1 mM PMSF, 1:100 protease inhibitor (Millipore Sigma), and 1:500 phosphatase inhibitor (Millipore Sigma). Subsequently, protein A/G-coupled control IgG or specific antibodies were incubated with the pre-cleared A2780 cell lysate supernatant overnight at 4°C. Finally, after being subjected to three washes (each for 10 min with 250 mL IP buffer), the resulting beads bearing the bound immunocomplexes were sent to Biotech-pack (Beijing) for mass spectrometric analysis. The mass spectrometry proteomics data resulting from this study have been deposited at the ProteomeXchange Consortium (http://proteomecentral.proteomexchange.org) via the iProX partner repository under the dataset identifier PXD046297.

**Tumor samples and normal tissues**

All tissue samples used in this study were obtained from the Second Affiliated Hospital of Nanjing Medical University. The samples included primary tumors, surgical margin tissues paired with the tumors, and normal tissues. To ensure accurate representation, these tissue samples underwent pathological and histological examination. Clinical and pathological data were collected to confirm that the percentage of tumor cells in the samples exceeded 70%. This study was conducted in compliance with the guidelines outlined in the Helsinki Declaration of 1975 and approved by the Institutional Ethics Committees of The Second Affiliated Hospital of Nanjing Medical University (approval notice no. [2023]-KY-007-01).

**Tissue microarrays and immunohistochemical staining**

Commercially available adult human normal ovary tissue arrays and human OC tissue arrays were purchased from Shanghai Wellbio Technology Co., Ltd. (Wellbio Technology Co., Shanghai, China). For immunohistochemistry, the tissue array chip was incubated with the primary antibody rabbit anti-BIN2-T423pS421p polyclonal antibody (Zoonbio BioTech, 1:100 dilution), followed by visualization using diaminobenzidine (DAB).

**Data mining for the prognostic value of BIN2 in OC**

The GEPIA database (http://gepia2.cancer-pku.cn/#analysis) and Kaplan–Meier Plotter database (http://kmplot.com/analysis/) were used to investigate the prognostic significance of BIN2 expression in human OC. The GEPIA database, an online platform, sources its analyzed tumor and normal tissue data from the TCGA database. The analysis of BIN2 expression in normal ovaries and OCs was conducted using the GEPIA database. Additionally, the Kaplan–Meier Plotter database was leveraged to ascertain the correlation between BIN2 expression and progression-free survival (PFS) in grade I and grade II OC.

**Statistical analysis**

All statistical graphs showing western blots or DNA gels were based on three independent repetitions, while those depicting blood biochemical indexes were obtained from seven independent repetitions. Each dot on the graphs represents one repetition. When the standard error of all individual data points in a group collected randomly was significantly smaller than the average value, the corresponding sample size was deemed appropriate and reliable. The mean ± SEM was used to present the data. For statistical comparisons between the two groups, the Student's t-test was employed using the Microsoft Excel program (Microsoft, Redmond, WA, USA) while Kruskal–Wallis one-way nonparametric ANOVA (Prism, GraphPad Software, San Diego, CA, USA) was used for multiple comparisons. P-values less than 0.05 were considered statistically significant.

**Supplementary tables**

**Supplementary table 1.**

**Plasmid construction primers for mouse *Bin2* and *Hdac1***

| Name | Sequence | Length |
| --- | --- | --- |
| SalI-Bin2-F in pFastBac-EGFP-StrepII | ACGCGTCGACTAGCAGAGGGCAAGGCGGGCGGAG | 1512bp |
| NotI-Bin2-R in pFastBac-EGFP-StrepII | AAGGAAAAAAGCGGCCGCGAGCTCCGTGTTTTCACTGGTAAATAC |
| Sal1-Bin2(BAR)-F in pFastBac-EGFP-StrepII | ACGCGTCGACTAGCAGAGGGCAAGGCGGGCGGAG | 717bp |
| Not1-Bin2(BAR)- R in pFastBac-EGFP-StrepII | AAGGAAAAAAGCGGCCGCTTTCTCCAGTTTGCTCATCAC |
| Sal1-Bin2(DUF)-F in pFastBac-EGFP-StrepII | ACGCGTCGACTACAACATTCCAACAAAGTCTTTG | 795bp |
| Not1-Bin2(DUF)-R in pFastBac-EGFP-StrepII | AAGGAAAAAAGCGGCCGCGAGCTCCGTGTTTTCACTGG |
| EcoRI-Strep-Bin2-F in pcDNA3.1&SK(+) | CCGGAATTCGCCACCATGTGGAGTCATCCACAGTTCGAGAAGCTTCTACTTCTACTTCTAGCAGAGGGCAAGGCGGGCGGAG | 2229bp |
| XhoI-EGFP-R in pcDNA3.1&SK(+) | CCGCTCGAGTTACTTGTACAGCTCGTCCATGC |
| EcoRI-hdac1-F in pGEX6p1 | CCGGAATTCATGGCGCAGACGCAGGGCAC | 1473bp |
| XhoI-StrepII -hdac1-R in pGEX6p1 | CCGCTCGAGTTACTTCTCGAACTGTGGATGACTCCAGGCCAACTTGACCTCTTCTTTG |
| EcoRI - Kozac -6xLeu -hdac1-F in pcDNA3.1 | CCGGAATTCGCCACCATGCTTCTACTTCTACTTCTAGCGCAGACTCAGGGCAC | 1449bp |
| EcorV-hdac1-R in pcDNA3.1 | CCGGATATCGGCCAACTTGACCTCTTCTTTG |
|  |  |
|  |  |  |
|  |  |

This table includes all primers for plasmid construction of mouse *Bin2* and *Hdac1.*

**Supplementary table 2. All insert & vector information and related figures.**

| Insert in plasmid | Related figures |
| --- | --- |
| *BIN2(TS-DD)*-*Egfp-StrepII* in pcDNA3.1(+) | Figure 2C |
| *HDAC1-Rfp-Flag* in pcDNA3.1(+) | Figure 2C |
| *BIN2-Egfp-StrepII* in pFastBac(+) | Figure 2J and 2L |
| *BIN2(TS-AA)*-*Egfp-StrepII* in pFastBac | Figure 2J and 2K |
| *HDAC1-strepII* in pGEX-6p1 | Figure 2K and 2L, Figure 2O and 2P |
| *BAR-Egfp-StrepII* in pFastBac(+) | Figure 2N and 2P |
| *DUF-Egfp-StrepII* in pFastBac(+) | Figure 2N and 2O |

**Supplementary table 3. RT-PCR primers for TSGs**

| Primer name | DNA templates (5′-3′) |
| --- | --- |
| Mbd4 - F | Oligo: CAGGAACAGAATGCCGTAAGT |
| Mbd4 - R | Oligo: CCTTGTGGGCTGATAAAGTACAC |
| Topors - F | Oligo: TCACCCCTGATCGACGATTTC |
| Topors - R | Oligo: CTATCCGGTGGAGTTGTTGTTC |
| Sh2b3 - F | Oligo: TTGAGATGCCTGACAACCTTTAC |
| Sh2b3 - R | Oligo: GCTCTAGGGCTGAGGGAATATG |
