# Supplementary material for: BIN2 inhibition suppress ovarian cancer progression meanwhile protect ovarian function through downregulating HDAC1 and RPS6 phosphorylation respectively
Source: Clin Transl Med. 2024 Oct 16;14(10):e70051. doi: 10.1002/ctm2.70051 (PMC11480968; doi:10.1002/ctm2.70051)

Supplementary immunohistochemistry figure 1

Related to figure 2R

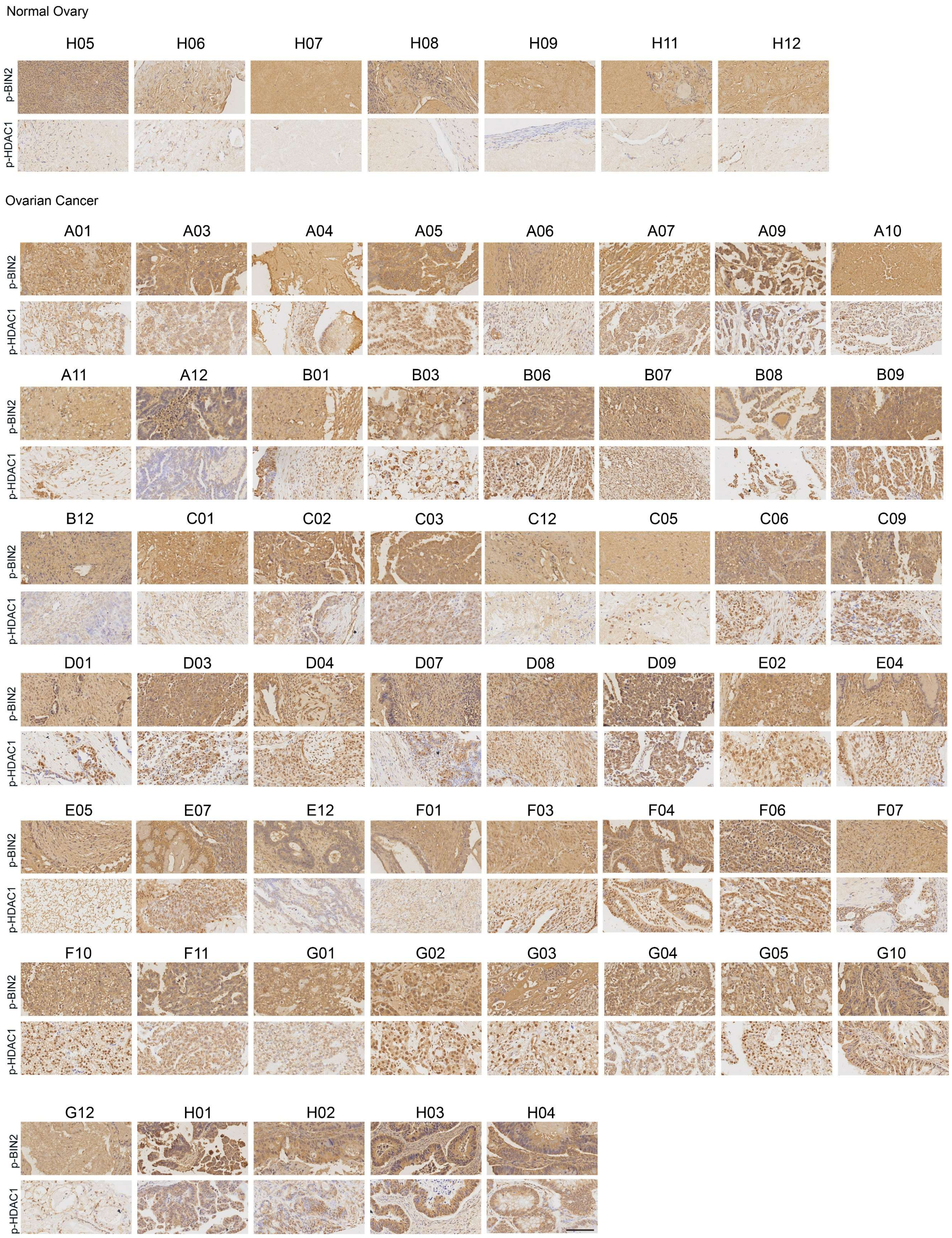

Supplementary immunohistochemistry figure 2

Related to supplementary figure 1D

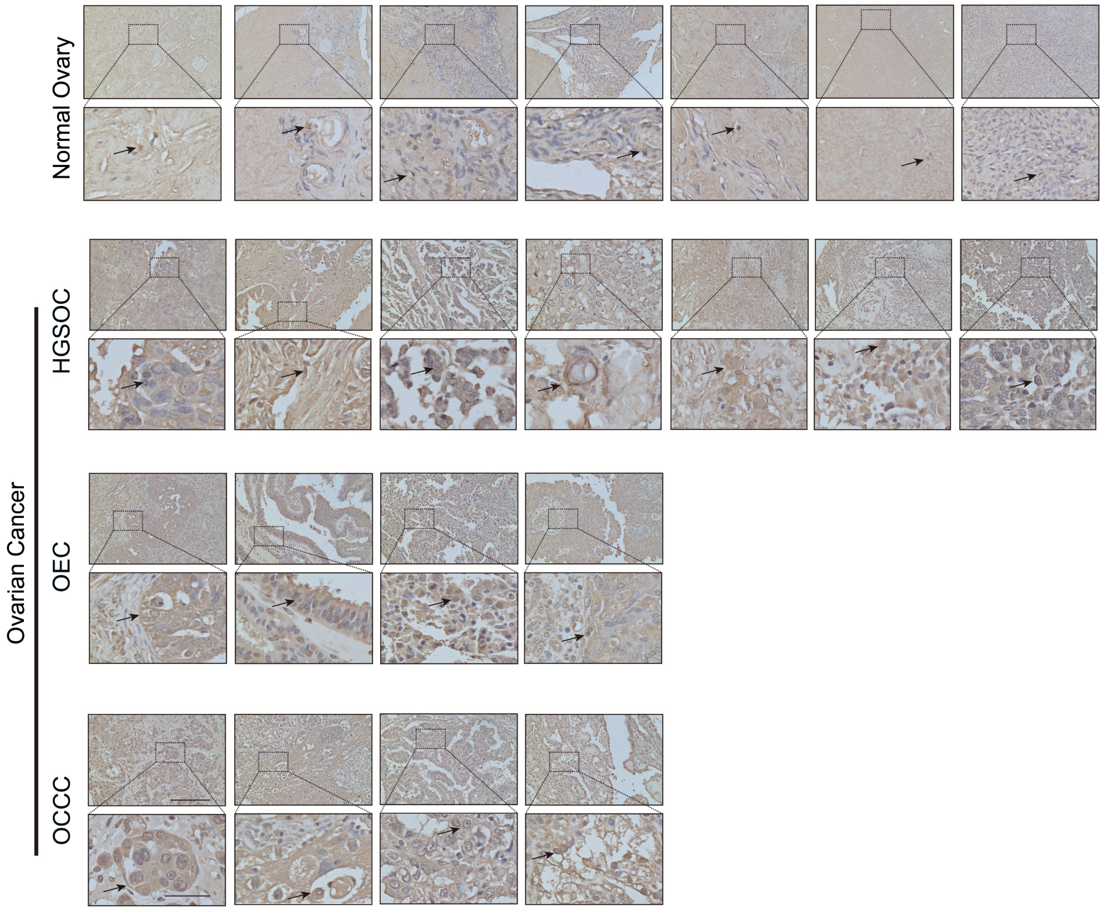

Supplementary immunohistochemistry figure 3

Related to supplementary figure 2A and 2C

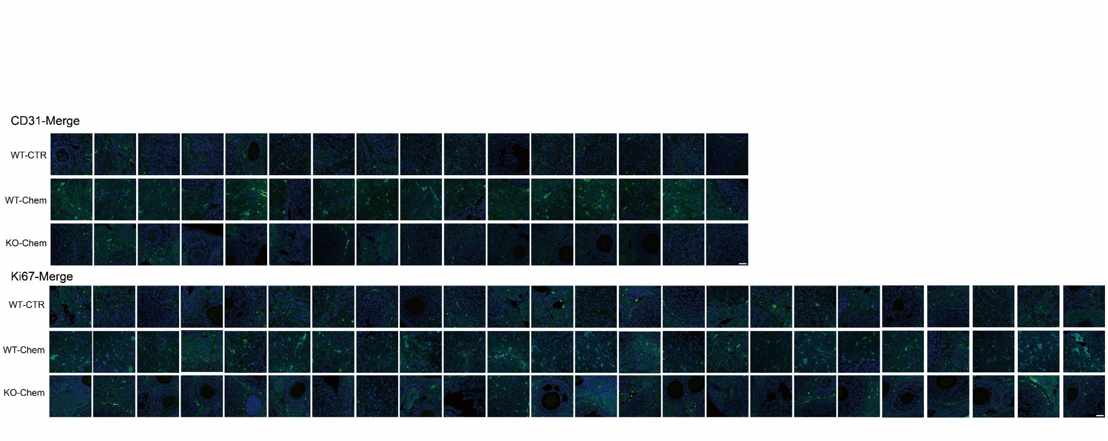

Supplementary immunohistochemistry figure 4

Related to supplementary figure 6D

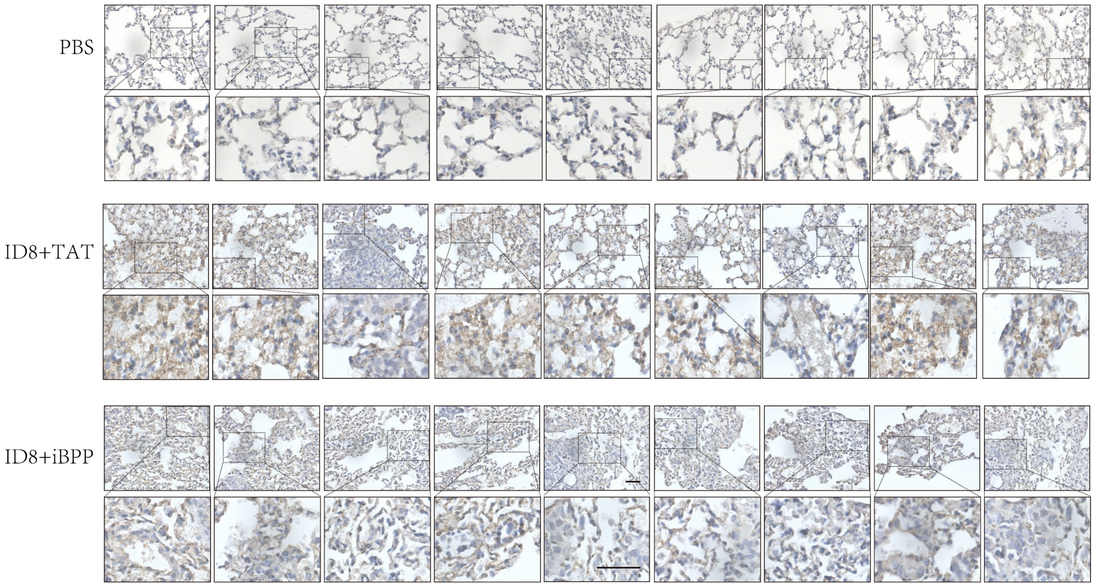

Supplement: Supplementary file 14 — Supporting Information [file CTM2-14-e70051-s003.pdf]
